# Investigating the role of mental health and lifestyle behaviours in the risk of adolescent suicidal behaviours through machine learning

**DOI:** 10.1371/journal.pone.0358758

**Published:** 2026-10-21

**Authors:** Yanmei Ding, Yanzhao Lei

**Affiliations:** 1 Shanghai Civil Aviation College, Shanghai, China; 2 Shanghai SIPO Polytechnic, Shanghai, China; Robert Gordon University, UNITED KINGDOM OF GREAT BRITAIN AND NORTHERN IRELAND

## Abstract

**Background:**

Suicidal behaviors among adolescents are a serious global public health concern and are closely linked to both mental health and everyday lifestyle behaviors. Suicidal ideation, planning, and attempts lie along a spectrum of increasing severity. However, how psychological distress and health-related behaviors interact to influence suicide risk is complex and not yet fully understood. This study aimed to identify key mental health and lifestyle factors associated with different stages of adolescent suicidal behaviors using interpretable machine learning methods.

**Methods:**

Data were obtained from the 2021 Youth Risk Behavior Surveillance System (YRBS), a nationally representative survey of U.S. high school students. After data cleaning and multiple imputation, 16,946 adolescents aged 14–18 years were included in the analysis. Extreme Gradient Boosting (XGBoost) models were developed to predict suicidal ideation, suicide planning, and suicide attempts. Model performance was assessed using the area under the receiver operating characteristic curve (AUC) and related metrics. Feature importance was examined using gain-based measures, while SHapley Additive exPlanations (SHAP) and partial dependence plots were used to explore the direction, strength, and nonlinear effects of key predictors.

**Results:**

All three XGBoost models showed satisfactory predictive performance. Mental health factors, particularly persistent feelings of sadness and self-rated mental health, were the strongest predictors across all stages of suicidal behaviors. Lifestyle behaviors, including sleep duration, physical activity, diet, screen time, and substance use, also contributed meaningfully to risk prediction, although their relative importance differed by outcome. SHAP analyses indicated that greater sadness and poorer mental health were strongly associated with higher suicide risk, whereas adequate sleep and higher levels of physical activity were associated with lower predicted risk. Substance use, including alcohol, cigarette, and marijuana use, played an important role in predicting suicide attempts than suicidal ideation or planning. Partial dependence analyses showed nonlinear and threshold effects, especially for sadness, mental health status, and sleep duration.

**Conclusion:**

Mental health factors, especially persistent sadness and overall psychological well-being, were among the most prominent factors associated with suicidal behaviors among adolescents, while lifestyle behaviors showed important and stage-specific associations. Interpretable machine learning approaches help identify complex and nonlinear patterns associated with adolescent suicide risk and offer valuable insights to guide targeted, evidence-based prevention efforts.

## 1 Introduction

Research on adolescent suicidal behaviours has expanded considerably in recent years, reflecting growing global concern about youth mental health. Suicide is now recognised as one of the leading causes of death among adolescents and young people worldwide, representing a substantial public health burden [1]. In the United States, suicide consistently ranks among the leading causes of mortality for individuals aged 10–19 years, with national surveillance data showing rising levels of suicidal ideation and attempt [2]. Adolescence is a critical developmental period marked by rapid biological, psychological, and social changes, during which exposure to adverse mental and behavioural factors may have long-lasting effects [3]. Therefore, identifying key factors associated with adolescent suicidal behaviours is essential for effective prevention and early intervention.

Suicidal behaviours are commonly understood as a continuum that includes suicidal ideation, suicide planning, and suicide attempts, with increasing severity and clinical significance across stages [4]. Suicidal ideation reflects early psychological vulnerability, while suicide planning and attempts signal heightened risk and a greater likelihood of serious outcomes, including suicide death [5]. Evidence suggests that progression along this continuum is shaped by the accumulation and interaction of multiple risk factors, rather than by isolated exposures [6].

In the literature, demographic and physical health characteristics are frequently examined as important contextual factors in studies of adolescent suicidal behaviours. For instance, consistent sex differences have been observed, with adolescent girls reporting higher rates of suicidal ideation and planning, while boys experience higher rates of suicide mortality [7,8]. In addition, age-related patterns have also been examined, with older adolescents generally showing higher prevalence of suicidal ideation and attempts, possibly reflecting increasing academic demands, social pressures, and emotional stress [9]. Furthermore, physical health and body weight status have likewise been associated with suicidality, highlighting the close interplay between physical and psychological health during adolescence [10,11].

Mental health factors play a central role in adolescent suicidal behaviours and are among the strongest predictors across all stages of the suicidal continuum. Persistent feelings of sadness or hopelessness, depressive symptoms, anxiety, and poor self-rated mental health have been consistently linked to suicidal ideation, planning, and attempt [12–14]. Adolescents experiencing emotional distress may have difficulties with emotion regulation, negative cognitive appraisals, and reduced coping capacity, all of which increase vulnerability to suicidal thoughts and behaviours [15]. In addition, psychosocial stressors such as sleep difficulties, perceived pressure, body image dissatisfaction, and limited social support have been shown to further exacerbate suicide risk [16,17]. Conversely, supportive peer relationships social connectedness appear to be protective, potentially buffering the harmful effects of psychological distress [18].

Lifestyle behaviours have also been increasingly recognised as important contributors to adolescent suicidal behaviours. Substance use, including alcohol consumption, cigarette smoking, marijuana use, and electronic vapor product use, has been consistently associated with increased risk of suicidal ideation and attempts [19–21]. These behaviours often co-occur with emotional distress and impulsivity, which may amplify psychological vulnerability. In addition, unhealthy lifestyle patterns, such as insufficient sleep, excessive screen time, low physical activity, and poor dietary habits, have been linked to poorer mental health and higher suicide risk among adolescents [22–25].

Despite a substantial body of research, most studies of adolescent suicidal behaviours rely on conventional regression-based approaches that typically assess risk factors individually. These methods may be limited in their ability to capture nonlinear relationships, threshold effects, and complex interactions between mental health and lifestyle behaviours [26]. In recent years, machine learning methods have gained increasing attention in mental health research due to their ability to model high-dimensional data and uncover complex patterns without strong parametric assumptions [27,28]. However, their application in suicide research has been constrained by concerns regarding interpretability and clinical relevance.

Recent advances in explainable artificial intelligence, particularly SHapley Additive exPlanations (SHAP), provide a theoretically grounded framework for interpreting complex machine learning models by quantifying the contribution, direction, and interaction of individual predictors [29]. Integrating machine learning with explainability techniques allows researchers to move beyond prediction accuracy and to generate actionable insights that are relevant for public health and prevention strategies.

Therefore, the present study aims to investigate the role of mental health and lifestyle behaviours in the risk of adolescent suicidal behaviours using machine learning methods. Using data from the 2021 Youth Risk Behavior Surveillance System (YRBS), we applied XGBoost models combined with SHAP and partial dependence analyses to identify key predictors of suicidal ideation, suicide planning, and suicide attempts, and to characterize their nonlinear effects and interactions. By adopting an interpretable machine learning framework, this study sought to provide a more comprehensive understanding of adolescent suicide risk and informing targeted, evidence-based prevention efforts.

## 2 Materials and methods

### 2.1 Study design and data source

This study used a cross-sectional design based on data from the 2021 Youth Risk Behavior Surveillance System (YRBS); a nationally representative survey conducted every two years by the U.S. Centers for Disease Control and Prevention (CDC). The YRBS collects information on a broad range of health-related behaviours among high school students in grades 9–12, including mental health status, substance use, physical activity, dietary behaviours, and injury-related outcomes such as suicidal behaviours. The survey employs a three-stage cluster sampling design to generate representative estimates of U.S. adolescents attending both public and private schools [30].

The 2021 YRBS was administered anonymously using a standardized, self-completed questionnaire. Ethical approval and data governance procedures were managed by the CDC, and informed consent was obtained in accordance with local and federal requirements. This study used publicly available, de-identified YRBS data; as such, no additional institutional review board approval was required. Data were accessed on 1^st^ October 2024. The authors had no access to information that could identify individual participants during or after data collection.

### 2.2 Outcome measures: Suicidal behaviours

Three binary outcome variables were defined to represent distinct stages along the adolescent suicidal behavior continuum. Suicidal ideation was assessed using self-reported serious consideration of attempting suicide during the past 12 months. Suicide planning was defined as having made a plan for how to attempt suicide within the same time frame. Suicide attempts were defined as one or more self-reported suicide attempts during the past 12 months. These outcome measures were derived from standardized items included in the Youth Risk Behavior Surveillance System and have been widely used in epidemiological studies to assess suicide risk among adolescents [5,30].

### 2.3 Predictor variables

Predictor variables were selected *a priori* based on prior literature and their availability within the YRBS dataset and included demographic characteristics, mental health indicators, and lifestyle and substance use behaviors.

#### 2.3.1 Demographic variables.

Demographic covariates included age, sex, and race/ethnicity. Age was analyzed as a continuous variable, and sex was coded as reported in the YRBS questionnaire. Race/ethnicity was grouped into four mutually exclusive categories, including White, Black or African American, Hispanic/Latino (including single- and multiple-Hispanic categories), and all other races (including American Indian/Alaska Native, Asian, Native Hawaiian or Other Pacific Islander, and multiple non-Hispanic categories), consistent with prior YRBS-based analyses [31].

#### 2.3.2 Mental health indicators.

Mental health indicators included sadness and self-rated mental health, both assessed using standardized YRBS items. Persistent feelings of sadness or hopelessness during the past 12 months were used as a proxy for emotional distress, a widely used non-clinical indicator of adolescent mental health [12,14]. Self-rated mental health was included as a global subjective assessment of psychological well-being.

#### 2.3.3 Lifestyle and substance use behaviours.

Lifestyle behaviors included sleep duration, screen time, moderate-to-vigorous physical activity (MVPA) days, and dietary behaviors (breakfast consumption, intake of fruits, vegetables, soda, milk, and fruit juice).

Substance use variables included alcohol consumption, cigarette smoking, marijuana use, electronic vapor product use, and pain medication misuse, all assessed using standardized YRBS items referring to lifetime or recent use.

### 2.4 Missing data handling and imputation

The original analytic sample consisted of 17,232 adolescents. Participants with missing values for age (n = 98, 0.6%) and sex (n = 264, 1.5%) were excluded from the initial analyses, resulting in a final sample of 16,946 participants.

Among the remaining 16,946 participants, 12.26% of all data values across the 13 variables subjected to imputation were missing, with variable-specific missingness ranging from 1.49% to 42.71%. Missingness was 12.10% for body weight status. For mental health indicators, missingness was 1.49% for persistent sadness or hopelessness and 25.63% for self-rated mental health. For lifestyle behaviours, missingness was 20.92% for sleep duration, 3.29% for physical activity, and 4.20% for screen time. For dietary behaviours, missingness was 6.69% for breakfast consumption and 26.23% for soda consumption. For substance use variables, missingness was 5.74% for alcohol use, 1.81% for cigarette smoking, 1.88% for marijuana use, 6.63% for electronic vapor product use, and 42.71% for pain medication misuse.

To address missing data in other variables, multiple imputation was conducted using Stata (17), under the assumption that data were missing at random. Age and sex were included as predictors in the imputation model, consistent with best practices in epidemiological research [32]. Twenty imputations were generated, and analyses were conducted using the 20th imputed dataset.

System-generated imputation variables were subsequently removed prior to analysis. Multiple imputation has been shown to reduce bias and improve statistical efficiency compared with complete-case analysis in large observational datasets [33].

### 2.5 Statistical analysis and machine learning modeling

All analyses were conducted using R version 4.4.0. Descriptive statistics were used to summarize the demographic, mental health, lifestyle, and suicidal behavior characteristics of the study population. The three suicidal behavior outcomes were recoded as binary variables: suicidal ideation, suicide planning, and suicide attempt. For suicide attempt, participants reporting one or more suicide attempts during the past 12 months were classified as having attempted suicide, whereas those reporting zero attempts were classified as not having attempted suicide.

For each outcome, the analytic dataset was randomly divided into a training set (70%) and a testing set (30%) using stratified sampling to preserve the outcome distribution. Extreme Gradient Boosting (XGBoost) models were fitted separately for suicidal ideation, suicide planning, and suicide attempt. XGBoost is a tree-based ensemble algorithm that is well suited for modeling nonlinear relationships and complex interactions in high-dimensional data [34]. Five-fold cross-validation was used within the training set to tune key hyperparameters, including tree depth, learning rate, minimum child weight, subsampling ratio, column subsampling ratio, and number of boosting rounds.

To assess whether XGBoost provided additional predictive value over a conventional statistical approach, multivariable logistic regression models including the same predictors were also fitted for each outcome. Model performance was evaluated in the independent testing set using the area under the receiver operating characteristic curve (AUC) with 95% confidence intervals, accuracy, sensitivity, specificity, F1 score, and balanced accuracy. Because the outcomes were imbalanced, classification thresholds were selected in the training set using the Youden index and then applied to the testing set.

### 2.6 Model interpretability

Feature importance was initially assessed using the gain metric, which quantifies the relative contribution of each predictor to model performance during tree splitting. To enhance interpretability, SHapley Additive exPlanations (SHAP) were calculated to estimate the direction and magnitude of each predictor’s contribution to individual predictions and overall model behavior [29].

In addition, partial dependence plots (PDPs) were generated for key predictors to visualize nonlinear associations between feature values and the predicted risk of suicidal behaviors. The combination of SHAP and PDP analyses provides complementary insights into both global and local model behavior, and has become increasingly adopted in mental health machine learning research [35].

## 3 Results

### 3.1 Descriptive characteristics of the study population

Table 1 presents the descriptive characteristics of the study sample. After data cleaning and imputation procedures, a total of 16,946 adolescents were included in the final analytic sample. In terms of age distribution, the largest group was 15-year-olds (25.8%), followed by 16-year-olds (25.0%) and 17-year-olds (22.9%), with males accounting for 51.9% of the sample. The racial/ethnic composition was predominantly White (54.4%), followed by Hispanic/Latino (19.2%), Black or African American (13.9%), and other racial/ethnic groups (12.5%). With respect to suicidal behaviors, 21.0% of adolescents reported suicidal ideation, 17.2% reported having made a suicide plan, and 11.0% reported at least one suicide attempt during the past 12 months. Detailed information regarding the study variables is provided in S1 Table.

**Table 1:** Characteristics of the study population.

Variables		Considered suicide		Total	Made a suicide plan		Total	Actually attempted suicide					Total
		Yes	No		Yes	No		0 times	1 time	2 or 3 times	4 or 5 times	6 or more times	
**Age**													
12 years old or younger	n	14	20	34	13	21	34	22	4	1	3	4	34
	%	41.2%	58.8%	100.0%	38.2%	61.8%	100.0%	64.7%	11.8%	2.9%	8.8%	11.8%	100.0%
13 years old	n	15	45	60	11	49	60	51	5	3	1	0	60
	%	25.0%	75.0%	100.0%	18.3%	81.7%	100.0%	85.0%	8.3%	5.0%	1.7%	0.0%	100.0%
14 years old	n	681	2671	3352	562	2790	3352	2960	220	130	21	21	3352
	%	20.3%	79.7%	100.0%	16.8%	83.2%	100.0%	88.3%	6.6%	3.9%	0.6%	0.6%	100.0%
15 years old	n	949	3427	4376	831	3545	4376	3859	309	147	27	34	4376
	%	21.7%	78.3%	100.0%	19.0%	81.0%	100.0%	88.2%	7.1%	3.4%	0.6%	0.8%	100.0%
16 years old	n	919	3317	4236	731	3505	4236	3795	255	127	30	29	4236
	%	21.7%	78.3%	100.0%	17.3%	82.7%	100.0%	89.6%	6.0%	3.0%	0.7%	0.7%	100.0%
17 years old	n	784	3090	3874	598	3276	3874	3497	210	111	28	28	3874
	%	20.2%	79.8%	100.0%	15.4%	84.6%	100.0%	90.3%	5.4%	2.9%	0.7%	0.7%	100.0%
18 years old or older	n	195	819	1014	161	853	1014	897	54	40	11	12	1014
	%	19.2%	80.8%	100.0%	15.9%	84.1%	100.0%	88.5%	5.3%	3.9%	1.1%	1.2%	100.0%
Total	n	3557	13389	16946	2907	14039	16946	15081	1057	559	121	128	16946
	%	21.0%	79.0%	100.0%	17.2%	82.8%	100.0%	89.0%	6.2%	3.3%	0.7%	0.8%	100.0%
		*X^2^* = 15.62, *df* = 6, *p* = 0.016			*X^2^* = 30.66, *df* = 6, *p* < 0.001			*X^2^* = 119.20, *df* = 6, *p* < 0.001					
**Sex**													
Female	n	2381	5764	8145	1930	6215	8145	6962	653	381	77	72	8145
	%	29.2%	70.8%	100.0%	23.7%	76.3%	100.0%	85.5%	8.0%	4.7%	0.9%	0.9%	100.0%
Male	n	1176	7625	8801	977	7824	8801	8119	404	178	44	56	8801
	%	13.4%	86.6%	100.0%	11.1%	88.9%	100.0%	92.3%	4.6%	2.0%	0.5%	0.6%	100.0%
Total	n	3557	13389	16946	2907	14039	16946	15081	1057	559	121	128	16946
	%	21.0%	79.0%	100.0%	17.2%	82.8%	100.0%	89.0%	6.2%	3.3%	0.7%	0.8%	100.0%
		*X^2^* = 642.45, *df* = 1, *p < 0.001*			*X^2^* = 472.14, *df* = 1, p < 0.001			*X^2^* = 207.06, *df* = 4, *p* < 0.001					
**Race**													
White	n	1918	7303	9221	1506	7715	9221	8338	524	258	51	50	9221
	%	20.8%	79.2%	100.0%	16.3%	83.7%	100.0%	90.4%	5.7%	2.8%	0.6%	0.5%	100.0%
Black or African American	n	478	1874	2352	403	1949	2352	2017	181	110	19	25	2352
	%	20.3%	79.7%	100.0%	17.1%	82.9%	100.0%	85.8%	7.7%	4.7%	0.8%	1.1%	100.0%
Hispanic/Latino	n	679	2578	3257	570	2687	3257	2865	233	97	30	32	3257
	%	20.8%	79.2%	100.0%	17.5%	82.5%	100.0%	88.0%	7.2%	3.0%	0.9%	1.0%	100.0%
All Other Races	n	482	1634	2116	428	1688	2116	1861	119	94	21	21	2116
	%	22.8%	77.2%	100.0%	20.2%	79.8%	100.0%	87.9%	5.6%	4.4%	1.0%	1.0%	100.0%
Total	n	3557	13389	16946	2907	14039	16946	15081	1057	559	121	128	16946
	%	21.0%	79.0%	100.0%	17.2%	82.8%	100.0%	89.0%	6.2%	3.3%	0.7%	0.8%	100.0%
		*X^2^* = 4.95, *df* = 3, *p =* 0.016			*X^2^* = 18.72, *df* = 3, *p* < 0.001			*X^2^* = 73.90, *df* = 12, *p* < 0.001					

### 3.2 Model performance for predicting suicidal behaviors

Separate XGBoost models were developed to predict three binary outcomes: suicidal ideation, suicide planning, and suicide attempts. All models demonstrated satisfactory discriminative performance. Receiver operating characteristic (ROC) curves indicated good classification ability across the three outcomes, with area under the curve (AUC) values exceeding commonly accepted thresholds for population-based prediction tasks (Table 2).

**Table 2:** Comparison of predictive performance between XGBoost and logistic regression models.

Items	Considered suicide		Made a suicide plan		Actually attempted suicide	
	XGBoost	LR	XGBoost	LR	XGBoost	LR
Optimal Threshold	0.2112	0.2293	0.1793	0.1281	0.1160	0.1278
Accuracy	0.7731	0.7801	0.7506	0.7290	0.7611	0.7443
AUC	0.8722	0.8685	0.8547	0.8499	0.8201	0.8056
AUC 95% CI (lower)	0.8616	0.8577	0.8423	0.8372	0.8014	0.7863
AUC 95% CI (upper)	0.8829	0.8794	0.8672	0.8627	0.8388	0.8250
Sensitivity	0.8493	0.8174	0.8396	0.8526	0.7352	0.6977
Specificity	0.7528	0.7702	0.7328	0.7043	0.7643	0.7501
F1	0.6112	0.6096	0.5290	0.5120	0.4035	0.3750
Balanced Accuracy	0.8010	0.7938	0.7862	0.7784	0.7497	0.7239

Compared with logistic regression, XGBoost showed slightly higher discriminative performance across all three outcomes. For suicidal ideation, the AUC was 0.8722 (95% CI: 0.8616–0.8829) for XGBoost and 0.8685 (95% CI: 0.8577–0.8794) for logistic regression. For suicide planning, the corresponding AUC values were 0.8547 (95% CI: 0.8423–0.8672) and 0.8499 (95% CI: 0.8372–0.8627), respectively. For suicide attempts, XGBoost achieved an AUC of 0.8201 (95% CI: 0.8014–0.8388), compared with 0.8056 (95% CI: 0.7863–0.8250) for logistic regression. Although the improvements in discrimination were relatively modest, particularly for suicidal ideation and planning, XGBoost provided additional flexibility for capturing nonlinear relationships and complex predictive patterns. Combined with SHAP and partial dependence analyses, the model enabled interpretable assessment of nonlinear associations that may not be fully captured by conventional linear approaches.

### 3.3 Feature importance for suicidal ideation

Fig 1 illustrates the relative importance and directional effects of predictors associated with suicidal ideation. Based on the Gain metric, sadness emerged as the most influential predictor, followed by overall mental health status, sleep duration, and lifestyle-related behaviors such as breakfast frequency, physical activity (MVPA days), and screen time (Fig 1a).

**Fig 1:**
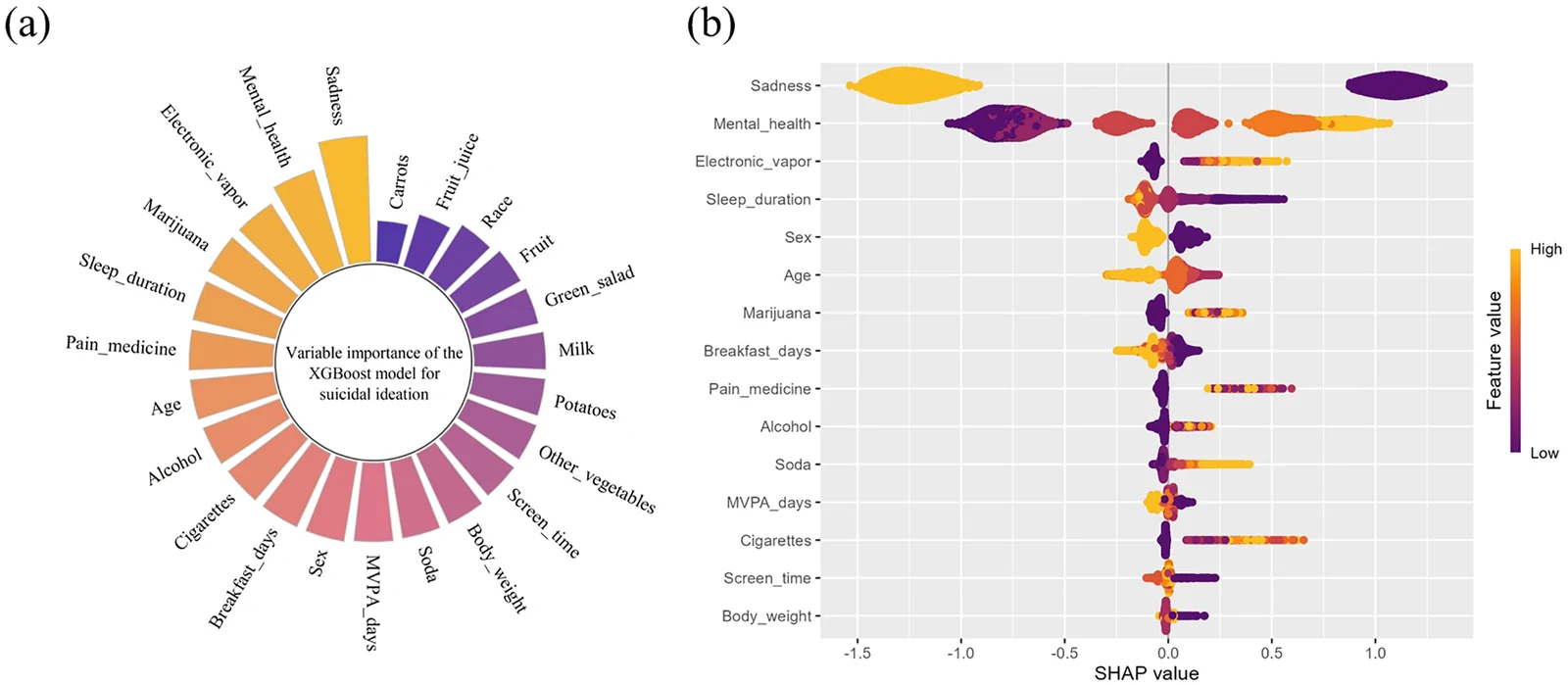
Feature importance and SHAP analysis for the prediction of suicidal ideation. Note: (a) Ranking of predictors based on the XGBoost Gain metric. (b) SHAP beeswarm plot illustrating the direction and magnitude of feature contributions to the risk of suicidal ideation.

SHAP analysis further revealed the directionality of these associations (Fig 1b). Higher levels of sadness and poorer mental health were strongly associated with increased risk of suicidal ideation. Shorter sleep duration and fewer breakfast days were also linked to higher predicted risk. In contrast, healthier dietary behaviors (e.g., fruit and milk consumption) and higher levels of physical activity were generally associated with lower SHAP values, indicating an association with lower predicted risk.

### 3.4 Feature importance for suicide planning

Fig 2 presents the results for suicide planning. Similar to suicidal ideation, sadness and overall mental health remained the dominant predictors of suicide planning. Sleep duration and age also contributed substantially to model performance.

**Fig 2:**
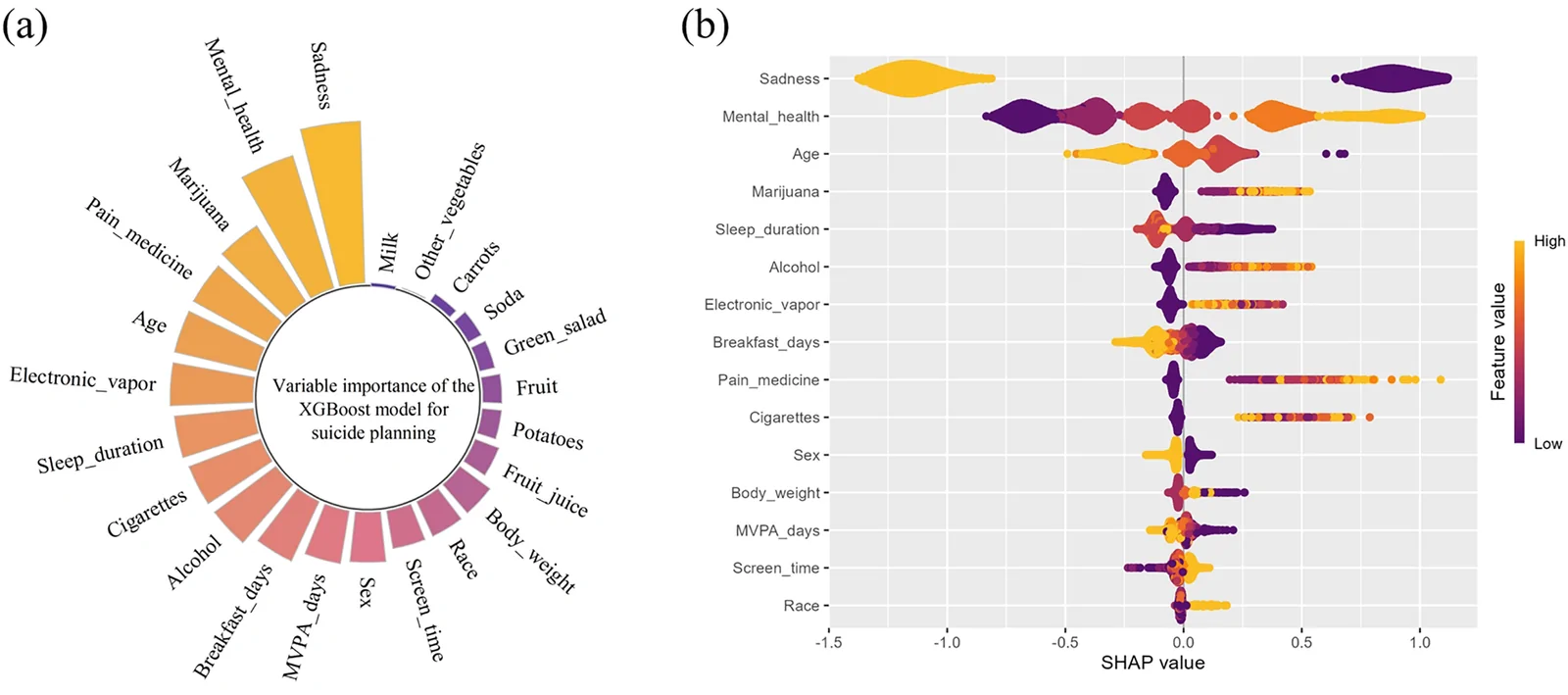
Feature importance and SHAP analysis for the prediction of suicide planning. Note: (a) Ranking of predictors based on the XGBoost Gain metric. (b) SHAP beeswarm plot illustrating the direction and magnitude of feature contributions to the risk of suicide planning.

SHAP values indicated that adolescents reporting higher levels of sadness and poorer mental health had markedly increased predicted probabilities of suicide planning. Additionally, lifestyle behaviors, including screen time, alcohol use, and electronic vapor product use, showed meaningful contributions, suggesting that behavioral risk accumulation may play an important role at the planning stage of suicidal behavior.

### 3.5 Feature importance for suicide attempt

Fig 3 displays the feature importance for suicide attempts. Compared with ideation and planning, mental health indicators remained central, but lifestyle behaviors showed relatively stronger contributions. In particular, substance-related variables (alcohol use, cigarette smoking, and marijuana use) ranked higher in importance for suicide attempts than for earlier stages of suicidal behavior.

**Fig 3:**
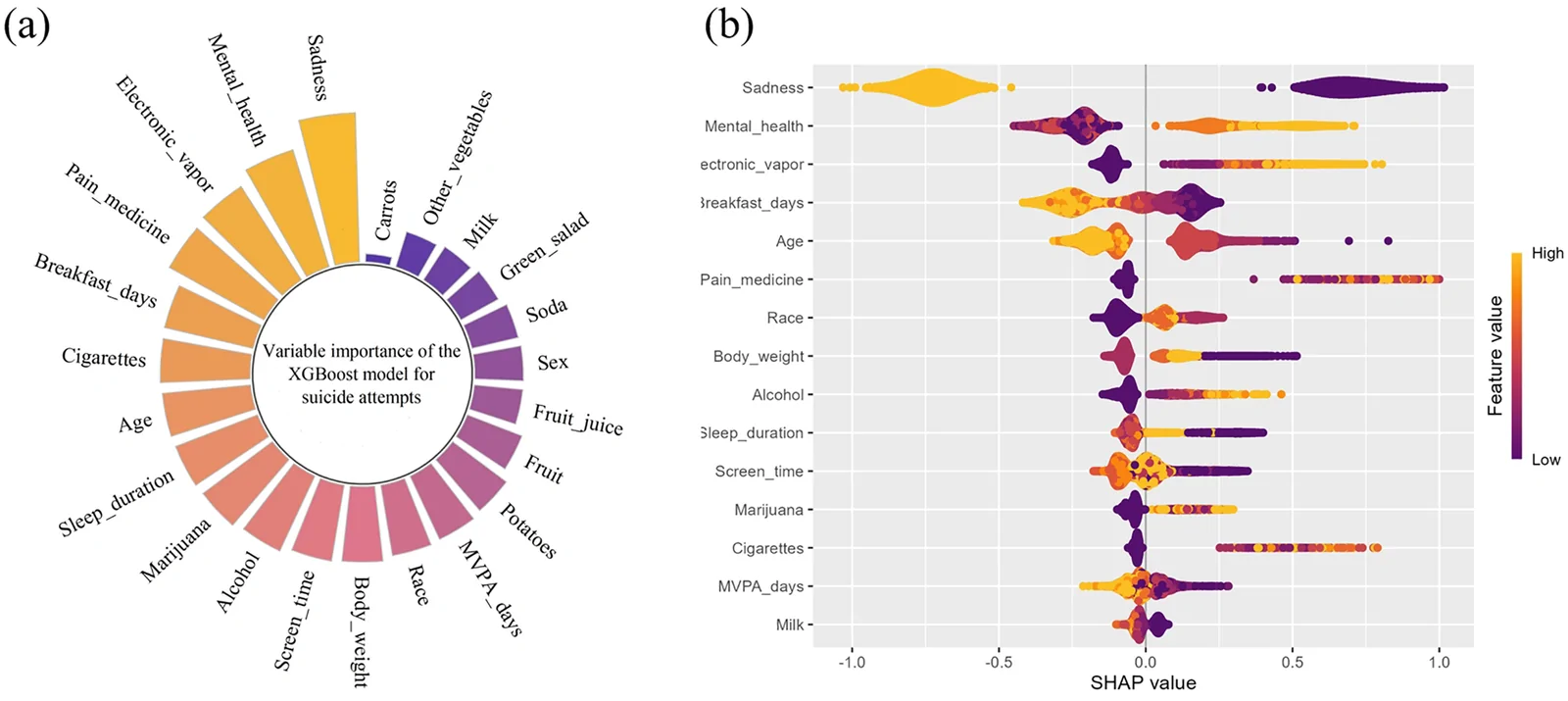
Feature importance and SHAP analysis for the prediction of suicide attempts. Note: (a) Ranking of predictors based on the XGBoost Gain metric. (b) SHAP beeswarm plot illustrating the direction and magnitude of feature contributions to the risk of suicide attempts.

SHAP analysis demonstrated that higher levels of substance use were associated with increased SHAP values, indicating elevated risk of suicide attempts. In contrast, healthier sleep duration and regular physical activity were associated with lower predicted risk.

### 3.6 Nonlinear effects of key predictors

Partial dependence and individual conditional expectation plots (Fig 4) were used to examine the nonlinear relationships between key predictors and suicidal behaviors. Sadness showed a monotonic positive association with suicidal risk, with the steepest increase observed at higher levels. Conversely, mental health exhibited a graded protective effect, with better self-rated mental health associated with progressively lower predicted risk.

**Fig 4:**
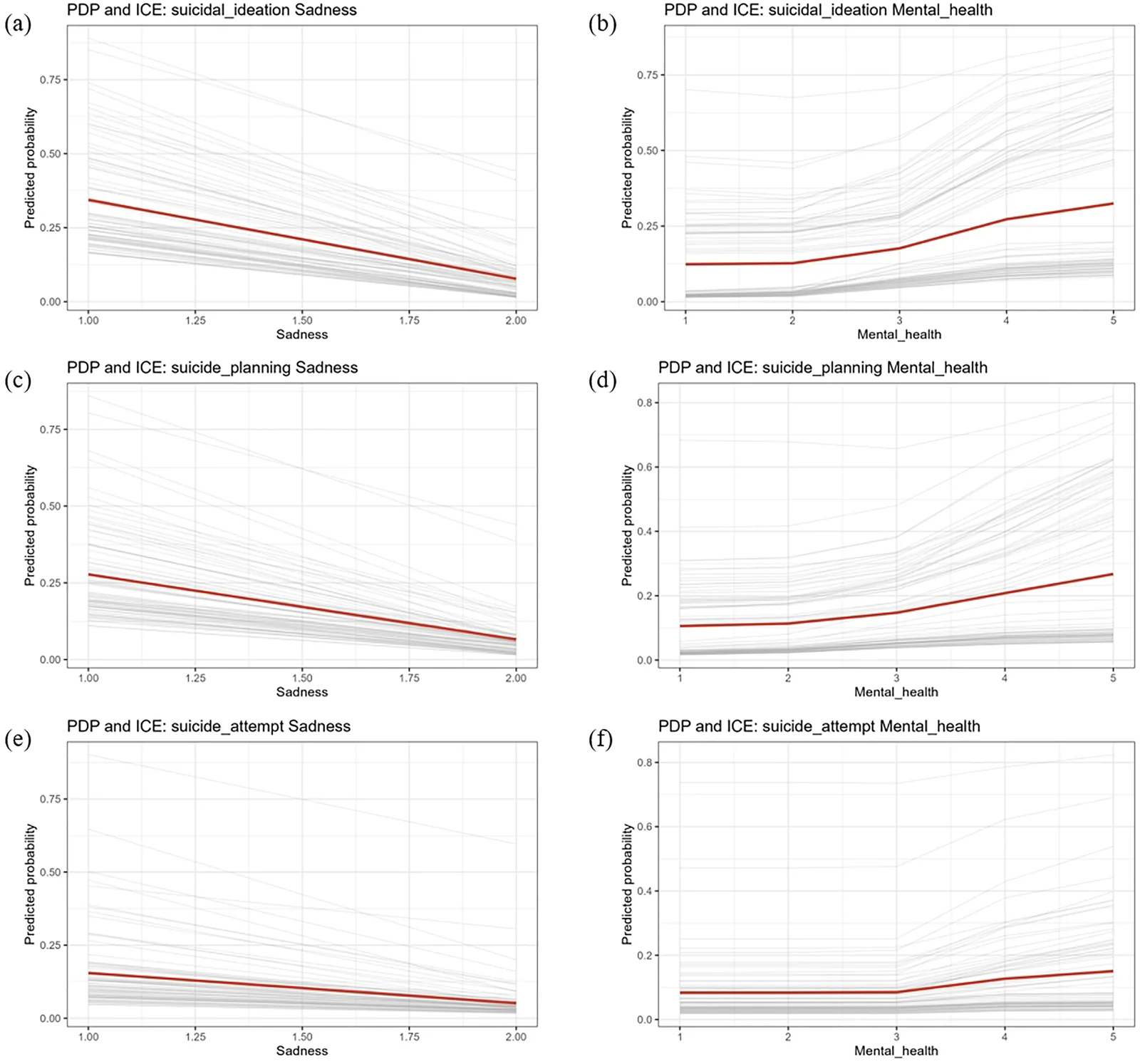
Partial dependence and individual conditional expectation plots of persistent sadness and self-rated mental health for suicidal behaviors.

Sleep duration demonstrated a nonlinear pattern, whereby insufficient sleep was associated with elevated suicidal risk, while moderate sleep duration was associated with the lowest predicted probability. These findings highlight the presence of threshold and saturation effects that may be missed using traditional linear modeling approaches.

## 4 Discussion

In this study, interpretable machine learning models were applied to investigate the roles of mental health and lifestyle behaviors in predicting three stages of adolescent suicidal behaviors, including suicidal ideation, suicide planning, and suicide attempts, using nationally representative data from the 2021 Youth Risk Behavior Surveillance System (YRBS). The XGBoost models demonstrated satisfactory predictive performance across all outcomes and enabled the identification of key predictors through Gain-based feature importance and SHapley Additive exPlanations (SHAP). This approach allowed for a comprehensive assessment of both the relative importance and the directional associations of predictors, as well as their nonlinear associations with suicidal behaviors.

Across all three outcomes, mental health indicators, particularly sadness and overall self-rated mental health, consistently emerged as the most influential predictors. Lifestyle behaviors, including sleep duration, physical activity, dietary habits, screen time, and substance use, also contributed meaningfully to risk prediction, with their relative importance varying across stages of the suicidal behavior continuum. Partial dependence analyses further revealed nonlinear and threshold effects, highlighting the added value of machine learning methods in capturing complex relationships that may be missed by traditional regression-based approaches [36,37].

### 4.1 Sociodemographic factors

Sociodemographic variables, including age, sex, and race/ethnicity, demonstrated comparatively smaller contributions to model performance relative to mental health and lifestyle factors. Age showed a consistent positive association with suicidal behaviors, with older adolescents exhibiting higher predicted risk. This finding aligns with prior epidemiological evidence suggesting that suicide risk increases across adolescence due to cumulative exposure to academic stress, social challenges, and developmental transitions [38].

Sex differences were also observed, although their overall importance was lower than that of psychological and behavioral predictors. Previous research has consistently reported higher prevalence of suicidal ideation and attempts among female adolescents, potentially reflecting sex-specific differences in emotional processing, internalizing symptoms, and exposure to social stress [39,40]. Race and ethnicity contributed modestly to prediction, suggesting that while structural and contextual inequities remain important, proximal mental health and behavioral factors may show stronger associations with suicide-related outcomes in population-based surveillance data.

### 4.2 Mental health factors

Mental health indicators showed the strongest associations with suicide-related outcomes across all three models. Sadness emerged as the single most influential predictor, particularly for suicidal ideation and suicide planning. SHAP analyses demonstrated a strong monotonic relationship, whereby higher levels of sadness were associated with sharply increased predicted risk. This finding is consistent with extensive literature linking depressive symptoms to suicidal thoughts and behaviors among adolescents [6,13].

Overall self-rated mental health showed a robust negative association with suicide-related outcomes. Adolescents reporting better mental health consistently exhibited lower predicted probabilities of suicidal ideation, planning, and attempts. Partial dependence plots suggested a graded relationship, indicating that higher levels of mental health were associated with lower predicted suicide risk. These findings highlight the importance of subjective mental health assessments, which capture dimensions of psychological well-being and resilience beyond the presence of discrete symptoms [41].

Notably, mental health variables remained highly influential even for suicide attempts, underscoring that psychological distress continues to play a central role throughout the suicidal behavior continuum. However, the nonlinear patterns observed suggest potential saturation effects at higher levels of distress, emphasizing the need for early identification and timely intervention.

### 4.3 Lifestyle and behavioral factors

Lifestyle behaviors demonstrated stage-specific associations with suicidal behaviors. Sleep duration showed a consistent nonlinear relationship, with insufficient sleep associated with elevated risk across all outcomes. Moderate sleep duration appeared to confer the lowest predicted risk, whereas short sleep duration was linked to substantially higher vulnerability. This finding aligns with prior evidence connecting sleep deprivation to emotional dysregulation, impaired coping, and increased suicidal risk among adolescents [24,42].

Physical activity and regular breakfast consumption generally exhibited negative associations with suicidal behaviors, particularly for suicidal ideation and planning. These behaviors may reflect broader health-promoting routines and psychosocial stability, which can buffer against emotional distress [43]. Dietary behaviors such as fruit and vegetable intake showed modest but consistent associations, suggesting that healthier eating patterns may serve as indirect indicators of overall well-being.

Substance-related behaviors, including alcohol use, cigarette smoking, marijuana use, and electronic vapor product use, were more prominent predictors of suicide attempts than of earlier stages. This pattern suggests that substance use was more strongly associated with suicide attempts than with earlier stages of suicidal behaviors. These associations may reflect the co-occurrence of substance use with related psychological and behavioral characteristics, such as impaired judgment, increased impulsivity, and reduced inhibition [20,21,44]. These findings highlight the importance of integrating substance use prevention into adolescent suicide prevention strategies.

### 4.4 Methodological strengths and limitations

This study has several notable strengths. First, it utilized a large, nationally representative dataset, enhancing the generalizability of the findings. Second, the application of interpretable machine learning methods enabled the identification of nonlinear relationships and complex interactions among predictors. The use of SHAP and partial dependence plots improved transparency and facilitated substantive interpretation of model outputs, addressing common concerns regarding the opaque nature of machine learning models [29].

Several limitations should be acknowledged. First, because the YRBS uses a cross-sectional design, temporality and causality cannot be established. Reverse causation remains possible because the relationship between mental health, suicidal behaviors, and lifestyle factors may be bidirectional. For example, psychological distress and suicidal behaviors may influence sleep patterns, physical activity levels, dietary behaviors, screen time, and substance use, rather than these lifestyle factors necessarily preceding suicidal behaviors. Therefore, the findings identified by logistic regression and XGBoost should be interpreted as predictive and associative relationships rather than causal effects. Second, although the models incorporated a broad range of predictor variables, including demographic characteristics, body weight status, mental health indicators, lifestyle behaviors, and substance use, residual confounding cannot be excluded. Important contextual and clinical factors, such as socioeconomic status, family environment, adverse childhood experiences, school climate, access to mental health services, and psychiatric comorbidities, were not comprehensively available in the 2021 YRBS dataset and therefore could not be included in the predictive models. These unmeasured factors may be associated with both lifestyle behaviors and mental health indicators, as well as suicidal behaviors, and may therefore have influenced the observed associations. Third, all measures were based on self-reported survey responses and may therefore be subject to recall bias, social desirability bias, and misclassification. Fourth, although machine learning methods can capture complex and nonlinear predictive patterns, the identified predictors should be interpreted as model-based associations rather than causal mechanisms. These findings may contribute to hypothesis generation and public health surveillance but should not be used for individual-level clinical decision-making without further validation.

### 4.5 Public health implications

The findings of this study highlight the central role of mental health, particularly sadness and overall psychological well-being, in adolescent suicidal behaviors, while also emphasizing the importance of lifestyle factors that may be relevant for risk assessment. School- and community-based interventions should prioritize early screening for emotional distress, promote healthy sleep and physical activity habits, and address substance use among adolescents. Importantly, the stage-specific patterns observed suggest that prevention strategies may need to be tailored across the suicidal behavior continuum, with greater emphasis on substance use prevention at later stages.

The application of interpretable machine learning provides a valuable framework for identifying high-risk adolescents and informing targeted suicide prevention efforts at the population level.

## 5 Conclusion

Using nationally representative data from the 2021 YRBS, this study identified key mental health and lifestyle factors associated with adolescent suicidal ideation, suicide planning, and suicide attempts through interpretable machine learning models. Sadness and overall mental health emerged as the most influential predictors across all outcomes, while lifestyle behaviors, particularly sleep duration and substance use, showed important, stage-specific associations. These findings highlight the complexity of adolescent suicidal behaviors and underscore the importance of integrated approaches that consider both psychological distress and health-related behaviors in suicide risk assessment and prevention research. Interpretable machine learning approaches offer a promising tool for advancing suicide prevention research and informing public health efforts.

## Supporting information

S1 Table Characteristics of all variables. (DOCX)

S1 File R code used for statistical analyses and machine learning modelling. (R)
