# Supplementary material for: Investigating the role of mental health and lifestyle behaviours in the risk of adolescent suicidal behaviours through machine learning
Source: PLoS One. 2026 Oct 21;21(10):e0358758. doi: 10.1371/journal.pone.0358758 (PMC13686503; doi:10.1371/journal.pone.0358758)
Supplement: Supplementary file 1 [file pone.0358758.s001.docx]

**Supplementary Table 1.** C**haracteristics of all variables.**

| **Variables** |  |  | **Considered suicide** | | **Total** |  | **Made a suicide plan** | | **Total** |  | **Actually attempted suicide** | | | | | **Total** |
| --- | --- | --- | --- | --- | --- | --- | --- | --- | --- | --- | --- | --- | --- | --- | --- | --- |
|  |  |  | **Yes** | **No** |  |  | **Yes** | **No** |  |  | **0 times** | **1 time** | **2 or 3 times** | **4 or 5 times** | **6 or more times** |  |
| **Age** |  |  |  |  |  |  |  |  |  |  |  |  |  |  |  |  |
| 12 years old or younger | n |  | 14 | 20 | 34 |  | 13 | 21 | 34 |  | 22 | 4 | 1 | 3 | 4 | 34 |
|  | % |  | 41.2% | 58.8% | 100.0% |  | 38.2% | 61.8% | 100.0% |  | 64.7% | 11.8% | 2.9% | 8.8% | 11.8% | 100.0% |
| 13 years old | n |  | 15 | 45 | 60 |  | 11 | 49 | 60 |  | 51 | 5 | 3 | 1 | 0 | 60 |
|  | % |  | 25.0% | 75.0% | 100.0% |  | 18.3% | 81.7% | 100.0% |  | 85.0% | 8.3% | 5.0% | 1.7% | 0.0% | 100.0% |
| 14 years old | n |  | 681 | 2671 | 3352 |  | 562 | 2790 | 3352 |  | 2960 | 220 | 130 | 21 | 21 | 3352 |
|  | % |  | 20.3% | 79.7% | 100.0% |  | 16.8% | 83.2% | 100.0% |  | 88.3% | 6.6% | 3.9% | 0.6% | 0.6% | 100.0% |
| 15 years old | n |  | 949 | 3427 | 4376 |  | 831 | 3545 | 4376 |  | 3859 | 309 | 147 | 27 | 34 | 4376 |
|  | % |  | 21.7% | 78.3% | 100.0% |  | 19.0% | 81.0% | 100.0% |  | 88.2% | 7.1% | 3.4% | 0.6% | 0.8% | 100.0% |
| 16 years old | n |  | 919 | 3317 | 4236 |  | 731 | 3505 | 4236 |  | 3795 | 255 | 127 | 30 | 29 | 4236 |
|  | % |  | 21.7% | 78.3% | 100.0% |  | 17.3% | 82.7% | 100.0% |  | 89.6% | 6.0% | 3.0% | 0.7% | .7% | 100.0% |
| 17 years old | n |  | 784 | 3090 | 3874 |  | 598 | 3276 | 3874 |  | 3497 | 210 | 111 | 28 | 28 | 3874 |
|  | % |  | 20.2% | 79.8% | 100.0% |  | 15.4% | 84.6% | 100.0% |  | 90.3% | 5.4% | 2.9% | 0.7% | .7% | 100.0% |
| 18 years old or older | n |  | 195 | 819 | 1014 |  | 161 | 853 | 1014 |  | 897 | 54 | 40 | 11 | 12 | 1014 |
|  | % |  | 19.2% | 80.8% | 100.0% |  | 15.9% | 84.1% | 100.0% |  | 88.5% | 5.3% | 3.9% | 1.1% | 1.2% | 100.0% |
| Total | n |  | 3557 | 13389 | 16946 |  | 2907 | 14039 | 16946 |  | 15081 | 1057 | 559 | 121 | 128 | 16946 |
|  | % |  | 21.0% | 79.0% | 100.0% |  | 17.2% | 82.8% | 100.0% |  | 89.0% | 6.2% | 3.3% | 0.7% | 0.8% | 100.0% |
|  |  |  | *X^2^* = 15.62, *df* = 6, *p* = 0.016 | | |  | *X^2^* = 30.66, *df* = 6, *p* < 0.001 | | |  | *X^2^* = 119.20, *df* = 6, *p* < 0.001 | | | | | |
| **Sex** |  |  |  |  |  |  |  |  |  |  |  |  |  |  |  |  |
| Female | n |  | 2381 | 5764 | 8145 |  | 1930 | 6215 | 8145 |  | 6962 | 653 | 381 | 77 | 72 | 8145 |
|  | % |  | 29.2% | 70.8% | 100.0% |  | 23.7% | 76.3% | 100.0% |  | 85.5% | 8.0% | 4.7% | 0.9% | 0.9% | 100.0% |
| Male | n |  | 1176 | 7625 | 8801 |  | 977 | 7824 | 8801 |  | 8119 | 404 | 178 | 44 | 56 | 8801 |
|  | % |  | 13.4% | 86.6% | 100.0% |  | 11.1% | 88.9% | 100.0% |  | 92.3% | 4.6% | 2.0% | 0.5% | 0.6% | 100.0% |
| Total | n |  | 3557 | 13389 | 16946 |  | 2907 | 14039 | 16946 |  | 15081 | 1057 | 559 | 121 | 128 | 16946 |
|  | % |  | 21.0% | 79.0% | 100.0% |  | 17.2% | 82.8% | 100.0% |  | 89.0% | 6.2% | 3.3% | 0.7% | 0.8% | 100.0% |
|  |  |  | *X^2^* = 642.45, *df* = 1, *p < 0.001* | | |  | *X^2^* = 472.14, *df* = 1, p < 0.001 | | |  | *X^2^* = 207.06, *df* = 4, p < 0.001 | | | | | |
| **Race** |  |  |  |  |  |  |  |  |  |  |  |  |  |  |  |  |
| White | n |  | 1918 | 7303 | 9221 |  | 1506 | 7715 | 9221 |  | 8338 | 524 | 258 | 51 | 50 | 9221 |
|  | % |  | 20.8% | 79.2% | 100.0% |  | 16.3% | 83.7% | 100.0% |  | 90.4% | 5.7% | 2.8% | 0.6% | 0.5% | 100.0% |
| Black or African American | n |  | 478 | 1874 | 2352 |  | 403 | 1949 | 2352 |  | 2017 | 181 | 110 | 19 | 25 | 2352 |
|  | % |  | 20.3% | 79.7% | 100.0% |  | 17.1% | 82.9% | 100.0% |  | 85.8% | 7.7% | 4.7% | 0.8% | 1.1% | 100.0% |
| Hispanic/Latino | n |  | 679 | 2578 | 3257 |  | 570 | 2687 | 3257 |  | 2865 | 233 | 97 | 30 | 32 | 3257 |
|  | % |  | 20.8% | 79.2% | 100.0% |  | 17.5% | 82.5% | 100.0% |  | 88.0% | 7.2% | 3.0% | 0.9% | 1.0% | 100.0% |
| All Other Races | n |  | 482 | 1634 | 2116 |  | 428 | 1688 | 2116 |  | 1861 | 119 | 94 | 21 | 21 | 2116 |
|  | % |  | 22.8% | 77.2% | 100.0% |  | 20.2% | 79.8% | 100.0% |  | 87.9% | 5.6% | 4.4% | 1.0% | 1.0% | 100.0% |
| Total | n |  | 3557 | 13389 | 16946 |  | 2907 | 14039 | 16946 |  | 15081 | 1057 | 559 | 121 | 128 | 16946 |
|  | % |  | 21.0% | 79.0% | 100.0% |  | 17.2% | 82.8% | 100.0% |  | 89.0% | 6.2% | 3.3% | 0.7% | 0.8% | 100.0% |
|  |  |  | *X^2^* = 4.95, *df* = 3, *p = 0.0*16 | | |  | *X^2^* = 18.72, *df* = 3, p < 0.001 | | |  | *X^2^* = 73.90, *df* = 12, p < 0.001 | | | | | |
| **Body Weight** |  |  |  |  |  |  |  |  |  |  |  |  |  |  |  |  |
| Underweight | n |  | 140 | 426 | 566 |  | 108 | 458 | 566 |  | 488 | 49 | 18 | 8 | 3 | 566 |
|  | % |  | 24.7% | 75.3% | 100.0% |  | 19.1% | 80.9% | 100.0% |  | 86.2% | 8.7% | 3.2% | 1.4% | 0.5% | 100.0% |
| Normal weight | n |  | 2096 | 8606 | 10702 |  | 1694 | 9008 | 10702 |  | 9690 | 583 | 303 | 60 | 66 | 10702 |
|  | % |  | 19.6% | 80.4% | 100.0% |  | 15.8% | 84.2% | 100.0% |  | 90.5% | 5.4% | 2.8% | 0.6% | 0.6% | 100.0% |
| Overweight | n |  | 634 | 2106 | 2740 |  | 510 | 2230 | 2740 |  | 2381 | 201 | 109 | 23 | 26 | 2740 |
|  | % |  | 23.1% | 76.9% | 100.0% |  | 18.6% | 81.4% | 100.0% |  | 86.9% | 7.3% | 4.0% | 0.8% | 0.9% | 100.0% |
| Obesity | n |  | 687 | 2251 | 2938 |  | 595 | 2343 | 2938 |  | 2522 | 224 | 129 | 30 | 33 | 2938 |
|  | % |  | 23.4% | 76.6% | 100.0% |  | 20.3% | 79.7% | 100.0% |  | 85.8% | 7.6% | 4.4% | 1.0% | 1.1% | 100.0% |
| Total | n |  | 3557 | 13389 | 16946 |  | 2907 | 14039 | 16946 |  | 15081 | 1057 | 559 | 121 | 128 | 16946 |
|  | % |  | 21.0% | 79.0% | 100.0% |  | 17.2% | 82.8% | 100.0% |  | 89.0% | 6.2% | 3.3% | 0.7% | 0.8% | 100.0% |
|  |  |  | *X^2^* = 35.30, *df* = 3, p < 0.001 | | |  | *X^2^* = 38.65, *df* = 3, p < 0.001 | | |  | *X^2^* = 81.57, *df* = 12, p < 0.001 | | | | | |
| **MVPA** |  |  |  |  |  |  |  |  |  |  |  |  |  |  |  |  |
| 0 days | n |  | 721 | 1932 | 2653 |  | 593 | 2060 | 2653 |  | 2294 | 196 | 119 | 23 | 21 | 2653 |
|  | % |  | 27.2% | 72.8% | 100.0% |  | 22.4% | 77.6% | 100.0% |  | 86.5% | 7.4% | 4.5% | 0.9% | 0.8% | 100.0% |
| 1 day | n |  | 282 | 891 | 1173 |  | 241 | 932 | 1173 |  | 996 | 109 | 50 | 9 | 9 | 1173 |
|  | % |  | 24.0% | 76.0% | 100.0% |  | 20.5% | 79.5% | 100.0% |  | 84.9% | 9.3% | 4.3% | 0.8% | 0.8% | 100.0% |
| 2 days | n |  | 425 | 1222 | 1647 |  | 332 | 1315 | 1647 |  | 1428 | 125 | 64 | 16 | 14 | 1647 |
|  | % |  | 25.8% | 74.2% | 100.0% |  | 20.2% | 79.8% | 100.0% |  | 86.7% | 7.6% | 3.9% | 1.0% | 0.9% | 100.0% |
| 3 days | n |  | 433 | 1464 | 1897 |  | 366 | 1531 | 1897 |  | 1660 | 133 | 71 | 18 | 15 | 1897 |
|  | % |  | 22.8% | 77.2% | 100.0% |  | 19.3% | 80.7% | 100.0% |  | 87.5% | 7.0% | 3.7% | 0.9% | 0.8% | 100.0% |
| 4 days | n |  | 386 | 1367 | 1753 |  | 316 | 1437 | 1753 |  | 1569 | 108 | 52 | 14 | 10 | 1753 |
|  | % |  | 22.0% | 78.0% | 100.0% |  | 18.0% | 82.0% | 100.0% |  | 89.5% | 6.2% | 3.0% | 0.8% | 0.6% | 100.0% |
| 5 days | n |  | 440 | 1802 | 2242 |  | 345 | 1897 | 2242 |  | 2022 | 134 | 56 | 12 | 18 | 2242 |
|  | % |  | 19.6% | 80.4% | 100.0% |  | 15.4% | 84.6% | 100.0% |  | 90.2% | 6.0% | 2.5% | 0.5% | 0.8% | 100.0% |
| 6 days | n |  | 201 | 1148 | 1349 |  | 162 | 1187 | 1349 |  | 1258 | 53 | 30 | 4 | 4 | 1349 |
|  | % |  | 14.9% | 85.1% | 100.0% |  | 12.0% | 88.0% | 100.0% |  | 93.3% | 3.9% | 2.2% | 0.3% | 0.3% | 100.0% |
| 7 days | n |  | 669 | 3563 | 4232 |  | 552 | 3680 | 4232 |  | 3854 | 199 | 117 | 25 | 37 | 4232 |
|  | % |  | 15.8% | 84.2% | 100.0% |  | 13.0% | 87.0% | 100.0% |  | 91.1% | 4.7% | 2.8% | 0.6% | 0.9% | 100.0% |
| Total | n |  | 3557 | 13389 | 16946 |  | 2907 | 14039 | 16946 |  | 15081 | 1057 | 559 | 121 | 128 | 16946 |
|  | % |  | 21.0% | 79.0% | 100.0% |  | 17.2% | 82.8% | 100.0% |  | 89.0% | 6.2% | 3.3% | 0.7% | 0.8% | 100.0% |
|  |  |  | *X^2^* = 197.02, *df* = 7, p < 0.001 | | |  | *X^2^* = 157.80, *df* = 7, p < 0.001 | | |  | *X^2^* = 114.14, *df* = 28, p < 0.001 | | | | | |
| **Screen time** |  |  |  |  |  |  |  |  |  |  |  |  |  |  |  |  |
| Less than 1 hour per day | n |  | 176 | 873 | 1049 |  | 141 | 908 | 1049 |  | 921 | 76 | 39 | 5 | 8 | 1049 |
|  | % |  | 16.8% | 83.2% | 100.0% |  | 13.4% | 86.6% | 100.0% |  | 87.8% | 7.2% | 3.7% | 0.5% | 0.8% | 100.0% |
| 1 hour per day | n |  | 112 | 716 | 828 |  | 84 | 744 | 828 |  | 749 | 50 | 20 | 5 | 4 | 828 |
|  | % |  | 13.5% | 86.5% | 100.0% |  | 10.1% | 89.9% | 100.0% |  | 90.5% | 6.0% | 2.4% | 0.6% | 0.5% | 100.0% |
| 2 hours per day | n |  | 349 | 1950 | 2299 |  | 283 | 2016 | 2299 |  | 2097 | 117 | 55 | 17 | 13 | 2299 |
|  | % |  | 15.2% | 84.8% | 100.0% |  | 12.3% | 87.7% | 100.0% |  | 91.2% | 5.1% | 2.4% | 0.7% | 0.6% | 100.0% |
| 3 hours per day | n |  | 560 | 2777 | 3337 |  | 443 | 2894 | 3337 |  | 3087 | 156 | 64 | 18 | 12 | 3337 |
|  | % |  | 16.8% | 83.2% | 100.0% |  | 13.3% | 86.7% | 100.0% |  | 92.5% | 4.7% | 1.9% | 0.5% | 0.4% | 100.0% |
| 4 hours per day | n |  | 598 | 2330 | 2928 |  | 466 | 2462 | 2928 |  | 2663 | 167 | 69 | 17 | 12 | 2928 |
|  | % |  | 20.4% | 79.6% | 100.0% |  | 15.9% | 84.1% | 100.0% |  | 90.9% | 5.7% | 2.4% | 0.6% | 0.4% | 100.0% |
| 5 or more hours per day | n |  | 1762 | 4743 | 6505 |  | 1490 | 5015 | 6505 |  | 5564 | 491 | 312 | 59 | 79 | 6505 |
|  | % |  | 27.1% | 72.9% | 100.0% |  | 22.9% | 77.1% | 100.0% |  | 85.5% | 7.5% | 4.8% | 0.9% | 1.2% | 100.0% |
| Total | n |  | 3557 | 13389 | 16946 |  | 2907 | 14039 | 16946 |  | 15081 | 1057 | 559 | 121 | 128 | 16946 |
|  | % |  | 21.0% | 79.0% | 100.0% |  | 17.2% | 82.8% | 100.0% |  | 89.0% | 6.2% | 3.3% | 0.7% | 0.8% | 100.0% |
|  |  |  | *X^2^* = 267.82, *df* = 5, p < 0.001 | | |  | *X^2^* = 266.65, *df* = 5, p < 0.001 | | |  | *X^2^* = 173.00, *df* = 20, p < 0.001 | | | | | |
| **Sleep** |  |  |  |  |  |  |  |  |  |  |  |  |  |  |  |  |
| 4 or less hours | n |  | 701 | 1026 | 1727 |  | 597 | 1130 | 1727 |  | 1305 | 188 | 134 | 40 | 60 | 1727 |
|  | % |  | 40.6% | 59.4% | 100.0% |  | 34.6% | 65.4% | 100.0% |  | 75.6% | 10.9% | 7.8% | 2.3% | 3.5% | 100.0% |
| 5 hours | n |  | 682 | 1694 | 2376 |  | 574 | 1802 | 2376 |  | 2044 | 175 | 109 | 28 | 20 | 2376 |
|  | % |  | 28.7% | 71.3% | 100.0% |  | 24.2% | 75.8% | 100.0% |  | 86.0% | 7.4% | 4.6% | 1.2% | 0.8% | 100.0% |
| 6 hours | n |  | 920 | 3240 | 4160 |  | 754 | 3406 | 4160 |  | 3709 | 283 | 132 | 21 | 15 | 4160 |
|  | % |  | 22.1% | 77.9% | 100.0% |  | 18.1% | 81.9% | 100.0% |  | 89.2% | 6.8% | 3.2% | .5% | 0.4% | 100.0% |
| 7 hours | n |  | 724 | 4008 | 4732 |  | 554 | 4178 | 4732 |  | 4389 | 218 | 99 | 15 | 11 | 4732 |
|  | % |  | 15.3% | 84.7% | 100.0% |  | 11.7% | 88.3% | 100.0% |  | 92.8% | 4.6% | 2.1% | 0.3% | 0.2% | 100.0% |
| 8 hours | n |  | 388 | 2588 | 2976 |  | 310 | 2666 | 2976 |  | 2758 | 142 | 58 | 9 | 9 | 2976 |
|  | % |  | 13.0% | 87.0% | 100.0% |  | 10.4% | 89.6% | 100.0% |  | 92.7% | 4.8% | 1.9% | 0.3% | 0.3% | 100.0% |
| 9 hours | n |  | 94 | 595 | 689 |  | 75 | 614 | 689 |  | 627 | 37 | 21 | 3 | 1 | 689 |
|  | % |  | 13.6% | 86.4% | 100.0% |  | 10.9% | 89.1% | 100.0% |  | 91.0% | 5.4% | 3.0% | 0.4% | 0.1% | 100.0% |
| 10 or more hours | n |  | 48 | 238 | 286 |  | 43 | 243 | 286 |  | 249 | 14 | 6 | 5 | 12 | 286 |
|  | % |  | 16.8% | 83.2% | 100.0% |  | 15.0% | 85.0% | 100.0% |  | 87.1% | 4.9% | 2.1% | 1.7% | 4.2% | 100.0% |
| Total | n |  | 3557 | 13389 | 16946 |  | 2907 | 14039 | 16946 |  | 15081 | 1057 | 559 | 121 | 128 | 16946 |
|  | % |  | 21.0% | 79.0% | 100.0% |  | 17.2% | 82.8% | 100.0% |  | 89.0% | 6.2% | 3.3% | 0.7% | 0.8% | 100.0% |
|  |  |  | *X^2^* = 719.83, *df* = 6, p < 0.001 | | |  | *X^2^* = 667.10, *df* = 6, p < 0.001 | | |  | *X^2^* = 649.35, *df* = 24, p < 0.001 | | | | | |
| **Breakfast** |  |  |  |  |  |  |  |  |  |  |  |  |  |  |  |  |
| 0 days | n |  | 1155 | 2583 | 3738 |  | 971 | 2767 | 3738 |  | 3081 | 329 | 222 | 46 | 60 | 3738 |
|  | % |  | 30.9% | 69.1% | 100.0% |  | 26.0% | 74.0% | 100.0% |  | 82.4% | 8.8% | 5.9% | 1.2% | 1.6% | 100.0% |
| 1 day | n |  | 438 | 1228 | 1666 |  | 378 | 1288 | 1666 |  | 1410 | 143 | 83 | 16 | 14 | 1666 |
|  | % |  | 26.3% | 73.7% | 100.0% |  | 22.7% | 77.3% | 100.0% |  | 84.6% | 8.6% | 5.0% | 1.0% | 0.8% | 100.0% |
| 2 days | n |  | 494 | 1719 | 2213 |  | 401 | 1812 | 2213 |  | 1944 | 158 | 82 | 20 | 9 | 2213 |
|  | % |  | 22.3% | 77.7% | 100.0% |  | 18.1% | 81.9% | 100.0% |  | 87.8% | 7.1% | 3.7% | 0.9% | 0.4% | 100.0% |
| 3 days | n |  | 375 | 1294 | 1669 |  | 307 | 1362 | 1669 |  | 1480 | 107 | 60 | 11 | 11 | 1669 |
|  | % |  | 22.5% | 77.5% | 100.0% |  | 18.4% | 81.6% | 100.0% |  | 88.7% | 6.4% | 3.6% | 0.7% | 0.7% | 100.0% |
| 4 days | n |  | 262 | 993 | 1255 |  | 205 | 1050 | 1255 |  | 1132 | 78 | 28 | 9 | 8 | 1255 |
|  | % |  | 20.9% | 79.1% | 100.0% |  | 16.3% | 83.7% | 100.0% |  | 90.2% | 6.2% | 2.2% | 0.7% | 0.6% | 100.0% |
| 5 days | n |  | 241 | 1096 | 1337 |  | 177 | 1160 | 1337 |  | 1246 | 64 | 22 | 3 | 2 | 1337 |
|  | % |  | 18.0% | 82.0% | 100.0% |  | 13.2% | 86.8% | 100.0% |  | 93.2% | 4.8% | 1.6% | 0.2% | 0.1% | 100.0% |
| 6 days | n |  | 134 | 747 | 881 |  | 109 | 772 | 881 |  | 831 | 36 | 11 | 1 | 2 | 881 |
|  | % |  | 15.2% | 84.8% | 100.0% |  | 12.4% | 87.6% | 100.0% |  | 94.3% | 4.1% | 1.2% | 0.1% | 0.2% | 100.0% |
| 7 days | n |  | 458 | 3729 | 4187 |  | 359 | 3828 | 4187 |  | 3957 | 142 | 51 | 15 | 22 | 4187 |
|  | % |  | 10.9% | 89.1% | 100.0% |  | 8.6% | 91.4% | 100.0% |  | 94.5% | 3.4% | 1.2% | 0.4% | 0.5% | 100.0% |
| Total | n |  | 3557 | 13389 | 16946 |  | 2907 | 14039 | 16946 |  | 15081 | 1057 | 559 | 121 | 128 | 16946 |
|  | % |  | 21.0% | 79.0% | 100.0% |  | 17.2% | 82.8% | 100.0% |  | 89.0% | 6.2% | 3.3% | 0.7% | 0.8% | 100.0% |
|  |  |  | *X^2^* = 534.01, *df* = 7, p < 0.001 | | |  | *X^2^* = 489.97, *df* = 7, p < 0.001 | | |  | *X^2^* = 426.45, *df* = 28, p < 0.001 | | | | | |
| **Fruit juice** |  |  |  |  |  |  |  |  |  |  |  |  |  |  |  |  |
| Did not drink fruit juice | n |  | 1248 | 4409 | 5657 |  | 1031 | 4626 | 5657 |  | 5060 | 350 | 175 | 36 | 36 | 5657 |
|  | % |  | 22.1% | 77.9% | 100.0% |  | 18.2% | 81.8% | 100.0% |  | 89.4% | 6.2% | 3.1% | 0.6% | 0.6% | 100.0% |
| 1 to 3 times | n |  | 1246 | 5197 | 6443 |  | 1017 | 5426 | 6443 |  | 5782 | 396 | 192 | 34 | 39 | 6443 |
|  | % |  | 19.3% | 80.7% | 100.0% |  | 15.8% | 84.2% | 100.0% |  | 89.7% | 6.1% | 3.0% | 0.5% | 0.6% | 100.0% |
| 4 to 6 times | n |  | 515 | 1712 | 2227 |  | 418 | 1809 | 2227 |  | 1945 | 146 | 87 | 30 | 19 | 2227 |
|  | % |  | 23.1% | 76.9% | 100.0% |  | 18.8% | 81.2% | 100.0% |  | 87.3% | 6.6% | 3.9% | 1.3% | 0.9% | 100.0% |
| 1 time per day | n |  | 162 | 691 | 853 |  | 117 | 736 | 853 |  | 765 | 55 | 22 | 5 | 6 | 853 |
|  | % |  | 19.0% | 81.0% | 100.0% |  | 13.7% | 86.3% | 100.0% |  | 89.7% | 6.4% | 2.6% | 0.6% | 0.7% | 100.0% |
| 2 times per day | n |  | 180 | 665 | 845 |  | 141 | 704 | 845 |  | 745 | 44 | 42 | 7 | 7 | 845 |
|  | % |  | 21.3% | 78.7% | 100.0% |  | 16.7% | 83.3% | 100.0% |  | 88.2% | 5.2% | 5.0% | 0.8% | 0.8% | 100.0% |
| 3 times per day | n |  | 80 | 281 | 361 |  | 70 | 291 | 361 |  | 316 | 24 | 13 | 5 | 3 | 361 |
|  | % |  | 22.2% | 77.8% | 100.0% |  | 19.4% | 80.6% | 100.0% |  | 87.5% | 6.6% | 3.6% | 1.4% | 0.8% | 100.0% |
| 4 or more times per day | n |  | 126 | 434 | 560 |  | 113 | 447 | 560 |  | 468 | 42 | 28 | 4 | 18 | 560 |
|  | % |  | 22.5% | 77.5% | 100.0% |  | 20.2% | 79.8% | 100.0% |  | 83.6% | 7.5% | 5.0% | 0.7% | 3.2% | 100.0% |
| Total | n |  | 3557 | 13389 | 16946 |  | 2907 | 14039 | 16946 |  | 15081 | 1057 | 559 | 121 | 128 | 16946 |
|  | % |  | 21.0% | 79.0% | 100.0% |  | 17.2% | 82.8% | 100.0% |  | 89.0% | 6.2% | 3.3% | 0.7% | 0.8% | 100.0% |
|  |  |  | *X^2^* = 23.80, *df* = 6, *p = 0.001* | | |  | *X^2^* = 29.26, *df* = 6, p < 0.001 | | |  | *X^2^* = 92.42, *df* = 28, p < 0.001 | | | | | |
| **Fruit** |  |  |  |  |  |  |  |  |  |  |  |  |  |  |  |  |
| Did not eat fruit | n |  | 586 | 1765 | 2351 |  | 477 | 1874 | 2351 |  | 2035 | 165 | 101 | 17 | 33 | 2351 |
|  | % |  | 24.9% | 75.1% | 100.0% |  | 20.3% | 79.7% | 100.0% |  | 86.6% | 7.0% | 4.3% | 0.7% | 1.4% | 100.0% |
| 1 to 3 times | n |  | 1288 | 4600 | 5888 |  | 1069 | 4819 | 5888 |  | 5223 | 404 | 191 | 44 | 26 | 5888 |
|  | % |  | 21.9% | 78.1% | 100.0% |  | 18.2% | 81.8% | 100.0% |  | 88.7% | 6.9% | 3.2% | 0.7% | 0.4% | 100.0% |
| 4 to 6 times | n |  | 735 | 2732 | 3467 |  | 607 | 2860 | 3467 |  | 3089 | 214 | 116 | 27 | 21 | 3467 |
|  | % |  | 21.2% | 78.8% | 100.0% |  | 17.5% | 82.5% | 100.0% |  | 89.1% | 6.2% | 3.3% | 0.8% | 0.6% | 100.0% |
| 1 time per day | n |  | 301 | 1499 | 1800 |  | 220 | 1580 | 1800 |  | 1659 | 93 | 31 | 8 | 9 | 1800 |
|  | % |  | 16.7% | 83.3% | 100.0% |  | 12.2% | 87.8% | 100.0% |  | 92.2% | 5.2% | 1.7% | 0.4% | 0.5% | 100.0% |
| 2 times per day | n |  | 328 | 1507 | 1835 |  | 250 | 1585 | 1835 |  | 1680 | 85 | 54 | 9 | 7 | 1835 |
|  | % |  | 17.9% | 82.1% | 100.0% |  | 13.6% | 86.4% | 100.0% |  | 91.6% | 4.6% | 2.9% | 0.5% | 0.4% | 100.0% |
| 3 times per day | n |  | 145 | 621 | 766 |  | 132 | 634 | 766 |  | 680 | 47 | 24 | 7 | 8 | 766 |
|  | % |  | 18.9% | 81.1% | 100.0% |  | 17.2% | 82.8% | 100.0% |  | 88.8% | 6.1% | 3.1% | 0.9% | 1.0% | 100.0% |
| 4 or more times per day | n |  | 174 | 665 | 839 |  | 152 | 687 | 839 |  | 715 | 49 | 42 | 9 | 24 | 839 |
|  | % |  | 20.7% | 79.3% | 100.0% |  | 18.1% | 81.9% | 100.0% |  | 85.2% | 5.8% | 5.0% | 1.1% | 2.9% | 100.0% |
| Total | n |  | 3557 | 13389 | 16946 |  | 2907 | 14039 | 16946 |  | 15081 | 1057 | 559 | 121 | 128 | 16946 |
|  | % |  | 21.0% | 79.0% | 100.0% |  | 17.2% | 82.8% | 100.0% |  | 89.0% | 6.2% | 3.3% | 0.7% | 0.8% | 100.0% |
|  |  |  | *X^2^* = 57.33, *df* = 6, p < 0.001 | | |  | *X^2^* = 68.17, *df* = 6, p < 0.001 | | |  | *X^2^* = 134.54, *df* = 24, p < 0.001 | | | | | |
| **Green salad** |  |  |  |  |  |  |  |  |  |  |  |  |  |  |  |  |
| Did not eat green salad | n |  | 1764 | 6244 | 8008 |  | 1453 | 6555 | 8008 |  | 7118 | 525 | 260 | 53 | 52 | 8008 |
|  | % |  | 22.0% | 78.0% | 100.0% |  | 18.1% | 81.9% | 100.0% |  | 88.9% | 6.6% | 3.2% | 0.7% | 0.6% | 100.0% |
| 1 to 3 times | n |  | 1178 | 4799 | 5977 |  | 952 | 5025 | 5977 |  | 5368 | 355 | 182 | 38 | 34 | 5977 |
|  | % |  | 19.7% | 80.3% | 100.0% |  | 15.9% | 84.1% | 100.0% |  | 89.8% | 5.9% | 3.0% | 0.6% | 0.6% | 100.0% |
| 4 to 6 times | n |  | 297 | 1117 | 1414 |  | 238 | 1176 | 1414 |  | 1255 | 87 | 46 | 12 | 14 | 1414 |
|  | % |  | 21.0% | 79.0% | 100.0% |  | 16.8% | 83.2% | 100.0% |  | 88.8% | 6.2% | 3.3% | 0.8% | 1.0% | 100.0% |
| 1 time per day | n |  | 176 | 734 | 910 |  | 147 | 763 | 910 |  | 802 | 52 | 38 | 7 | 11 | 910 |
|  | % |  | 19.3% | 80.7% | 100.0% |  | 16.2% | 83.8% | 100.0% |  | 88.1% | 5.7% | 4.2% | 0.8% | 1.2% | 100.0% |
| 2 times per day | n |  | 65 | 252 | 317 |  | 53 | 264 | 317 |  | 270 | 22 | 18 | 4 | 3 | 317 |
|  | % |  | 20.5% | 79.5% | 100.0% |  | 16.7% | 83.3% | 100.0% |  | 85.2% | 6.9% | 5.7% | 1.3% | 0.9% | 100.0% |
| 3 times per day | n |  | 24 | 93 | 117 |  | 20 | 97 | 117 |  | 101 | 6 | 5 | 3 | 2 | 117 |
|  | % |  | 20.5% | 79.5% | 100.0% |  | 17.1% | 82.9% | 100.0% |  | 86.3% | 5.1% | 4.3% | 2.6% | 1.7% | 100.0% |
| 4 or more times per day | n |  | 53 | 150 | 203 |  | 44 | 159 | 203 |  | 167 | 10 | 10 | 4 | 12 | 203 |
|  | % |  | 26.1% | 73.9% | 100.0% |  | 21.7% | 78.3% | 100.0% |  | 82.3% | 4.9% | 4.9% | 2.0% | 5.9% | 100.0% |
| Total | n |  | 3557 | 13389 | 16946 |  | 2907 | 14039 | 16946 |  | 15081 | 1057 | 559 | 121 | 128 | 16946 |
|  | % |  | 21.0% | 79.0% | 100.0% |  | 17.2% | 82.8% | 100.0% |  | 89.0% | 6.2% | 3.3% | 0.7% | 0.8% | 100.0% |
|  |  |  | *X^2^* = 15.88, *df* = 6, *p = 0.014* | | |  | *X^2^* = 15.56, *df* = 6, *p = 0.0*16 | | |  | *X^2^* = 109.68, *df* = 24, p < 0.001 | | | | | |
| **Potato** |  |  |  |  |  |  |  |  |  |  |  |  |  |  |  |  |
| Did not eat potatoes | n |  | 1496 | 5341 | 6837 |  | 1234 | 5603 | 6837 |  | 6075 | 456 | 220 | 44 | 42 | 6837 |
|  | % |  | 21.9% | 78.1% | 100.0% |  | 18.0% | 82.0% | 100.0% |  | 88.9% | 6.7% | 3.2% | 0.6% | 0.6% | 100.0% |
| 1 to 3 times | n |  | 1526 | 6024 | 7550 |  | 1211 | 6339 | 7550 |  | 6794 | 439 | 236 | 44 | 37 | 7550 |
|  | % |  | 20.2% | 79.8% | 100.0% |  | 16.0% | 84.0% | 100.0% |  | 90.0% | 5.8% | 3.1% | 0.6% | 0.5% | 100.0% |
| 4 to 6 times | n |  | 296 | 1127 | 1423 |  | 251 | 1172 | 1423 |  | 1264 | 89 | 46 | 12 | 12 | 1423 |
|  | % |  | 20.8% | 79.2% | 100.0% |  | 17.6% | 82.4% | 100.0% |  | 88.8% | 6.3% | 3.2% | 0.8% | 0.8% | 100.0% |
| 1 time per day | n |  | 115 | 453 | 568 |  | 99 | 469 | 568 |  | 487 | 41 | 19 | 11 | 10 | 568 |
|  | % |  | 20.2% | 79.8% | 100.0% |  | 17.4% | 82.6% | 100.0% |  | 85.7% | 7.2% | 3.3% | 1.9% | 1.8% | 100.0% |
| 2 times per day | n |  | 50 | 200 | 250 |  | 46 | 204 | 250 |  | 212 | 13 | 15 | 3 | 7 | 250 |
|  | % |  | 20.0% | 80.0% | 100.0% |  | 18.4% | 81.6% | 100.0% |  | 84.8% | 5.2% | 6.0% | 1.2% | 2.8% | 100.0% |
| 3 times per day | n |  | 20 | 79 | 99 |  | 16 | 83 | 99 |  | 81 | 6 | 9 | 2 | 1 | 99 |
|  | % |  | 20.2% | 79.8% | 100.0% |  | 16.2% | 83.8% | 100.0% |  | 81.8% | 6.1% | 9.1% | 2.0% | 1.0% | 100.0% |
| 4 or more times per day | n |  | 54 | 165 | 219 |  | 50 | 169 | 219 |  | 168 | 13 | 14 | 5 | 19 | 219 |
|  | % |  | 24.7% | 75.3% | 100.0% |  | 22.8% | 77.2% | 100.0% |  | 76.7% | 5.9% | 6.4% | 2.3% | 8.7% | 100.0% |
| Total | n |  | 3557 | 13389 | 16946 |  | 2907 | 14039 | 16946 |  | 15081 | 1057 | 559 | 121 | 128 | 16946 |
|  | % |  | 21.0% | 79.0% | 100.0% |  | 17.2% | 82.8% | 100.0% |  | 89.0% | 6.2% | 3.3% | 0.7% | 0.8% | 100.0% |
|  |  |  | *X^2^* = 8.21, *df* = 6, *p = .223* | | |  | *X^2^* = 16.022, *df* = 6, *p = 0.0*14 | | |  | *X^2^* = 272.32, *df* = 24, p < 0.001 | | | | | |
| **Carrot** |  |  |  |  |  |  |  |  |  |  |  |  |  |  |  |  |
| Did not eat Carrots | n |  | 2082 | 7410 | 9492 |  | 1701 | 7791 | 9492 |  | 8393 | 615 | 344 | 71 | 69 | 9492 |
|  | % |  | 21.9% | 78.1% | 100.0% |  | 17.9% | 82.1% | 100.0% |  | 88.4% | 6.5% | 3.6% | 0.7% | 0.7% | 100.0% |
| 1 to 3 times | n |  | 1086 | 4454 | 5540 |  | 866 | 4674 | 5540 |  | 5015 | 323 | 145 | 31 | 26 | 5540 |
|  | % |  | 19.6% | 80.4% | 100.0% |  | 15.6% | 84.4% | 100.0% |  | 90.5% | 5.8% | 2.6% | 0.6% | 0.5% | 100.0% |
| 4 to 6 times | n |  | 189 | 796 | 985 |  | 161 | 824 | 985 |  | 880 | 57 | 32 | 8 | 8 | 985 |
|  | % |  | 19.2% | 80.8% | 100.0% |  | 16.3% | 83.7% | 100.0% |  | 89.3% | 5.8% | 3.2% | 0.8% | 0.8% | 100.0% |
| 1 time per day | n |  | 97 | 390 | 487 |  | 81 | 406 | 487 |  | 438 | 23 | 19 | 3 | 4 | 487 |
|  | % |  | 19.9% | 80.1% | 100.0% |  | 16.6% | 83.4% | 100.0% |  | 89.9% | 4.7% | 3.9% | 0.6% | 0.8% | 100.0% |
| 2 times per day | n |  | 43 | 168 | 211 |  | 39 | 172 | 211 |  | 180 | 18 | 7 | 2 | 4 | 211 |
|  | % |  | 20.4% | 79.6% | 100.0% |  | 18.5% | 81.5% | 100.0% |  | 85.3% | 8.5% | 3.3% | 0.9% | 1.9% | 100.0% |
| 3 times per day | n |  | 21 | 62 | 83 |  | 21 | 62 | 83 |  | 68 | 6 | 5 | 2 | 2 | 83 |
|  | % |  | 25.3% | 74.7% | 100.0% |  | 25.3% | 74.7% | 100.0% |  | 81.9% | 7.2% | 6.0% | 2.4% | 2.4% | 100.0% |
| 4 or more times per day | n |  | 39 | 109 | 148 |  | 38 | 110 | 148 |  | 107 | 15 | 7 | 4 | 15 | 148 |
|  | % |  | 26.4% | 73.6% | 100.0% |  | 25.7% | 74.3% | 100.0% |  | 72.3% | 10.1% | 4.7% | 2.7% | 10.1% | 100.0% |
| Total | n |  | 3557 | 13389 | 16946 |  | 2907 | 14039 | 16946 |  | 15081 | 1057 | 559 | 121 | 128 | 16946 |
|  | % |  | 21.0% | 79.0% | 100.0% |  | 17.2% | 82.8% | 100.0% |  | 89.0% | 6.2% | 3.3% | 0.7% | 0.8% | 100.0% |
|  |  |  | *X^2^* = 17.34, *df* = 6, *p = 0.008* | | |  | *X^2^* = 25.20, *df* = 6, *p* < .000 | | |  | *X^2^* = 230.56, *df* = 24, p < 0.001 | | | | | |
| **Other vegetable** |  |  |  |  |  |  |  |  |  |  |  |  |  |  |  |  |
| Did not eat other vegetables | n |  | 764 | 2660 | 3424 |  | 645 | 2779 | 3424 |  | 2979 | 241 | 147 | 24 | 33 | 3424 |
|  | % |  | 22.3% | 77.7% | 100.0% |  | 18.8% | 81.2% | 100.0% |  | 87.0% | 7.0% | 4.3% | 0.7% | 1.0% | 100.0% |
| 1 to 3 times | n |  | 1337 | 4946 | 6283 |  | 1089 | 5194 | 6283 |  | 5621 | 387 | 201 | 40 | 34 | 6283 |
|  | % |  | 21.3% | 78.7% | 100.0% |  | 17.3% | 82.7% | 100.0% |  | 89.5% | 6.2% | 3.2% | 0.6% | 0.5% | 100.0% |
| 4 to 6 times | n |  | 779 | 2992 | 3771 |  | 621 | 3150 | 3771 |  | 3379 | 218 | 119 | 31 | 24 | 3771 |
|  | % |  | 20.7% | 79.3% | 100.0% |  | 16.5% | 83.5% | 100.0% |  | 89.6% | 5.8% | 3.2% | 0.8% | 0.6% | 100.0% |
| 1 time per day | n |  | 310 | 1354 | 1664 |  | 254 | 1410 | 1664 |  | 1508 | 97 | 39 | 11 | 9 | 1664 |
|  | % |  | 18.6% | 81.4% | 100.0% |  | 15.3% | 84.7% | 100.0% |  | 90.6% | 5.8% | 2.3% | 0.7% | 0.5% | 100.0% |
| 2 times per day | n |  | 200 | 797 | 997 |  | 152 | 845 | 997 |  | 895 | 66 | 27 | 2 | 7 | 997 |
|  | % |  | 20.1% | 79.9% | 100.0% |  | 15.2% | 84.8% | 100.0% |  | 89.8% | 6.6% | 2.7% | 0.2% | 0.7% | 100.0% |
| 3 times per day | n |  | 91 | 331 | 422 |  | 72 | 350 | 422 |  | 369 | 30 | 13 | 6 | 4 | 422 |
|  | % |  | 21.6% | 78.4% | 100.0% |  | 17.1% | 82.9% | 100.0% |  | 87.4% | 7.1% | 3.1% | 1.4% | 0.9% | 100.0% |
| 4 or more times per day | n |  | 76 | 309 | 385 |  | 74 | 311 | 385 |  | 330 | 18 | 13 | 7 | 17 | 385 |
|  | % |  | 19.7% | 80.3% | 100.0% |  | 19.2% | 80.8% | 100.0% |  | 85.7% | 4.7% | 3.4% | 1.8% | 4.4% | 100.0% |
| Total | n |  | 3557 | 13389 | 16946 |  | 2907 | 14039 | 16946 |  | 15081 | 1057 | 559 | 121 | 128 | 16946 |
|  | % |  | 21.0% | 79.0% | 100.0% |  | 17.2% | 82.8% | 100.0% |  | 89.0% | 6.2% | 3.3% | 0.7% | 0.8% | 100.0% |
|  |  |  | *X^2^* = 10.74, *df* = 6, *p = 0.097* | | |  | *X^2^* = 16.11, *df* = 6, *p* < .013 | | |  | *X^2^* = 117.41, *df* = 24, p < 0.001 | | | | | |
| **No soda** |  |  |  |  |  |  |  |  |  |  |  |  |  |  |  |  |
| Did not drink soda or pop | n |  | 932 | 4094 | 5026 |  | 751 | 4275 | 5026 |  | 4557 | 278 | 134 | 31 | 26 | 5026 |
|  | % |  | 18.5% | 81.5% | 100.0% |  | 14.9% | 85.1% | 100.0% |  | 90.7% | 5.5% | 2.7% | 0.6% | 0.5% | 100.0% |
| 1 to 3 times | n |  | 1297 | 5439 | 6736 |  | 1072 | 5664 | 6736 |  | 6088 | 398 | 193 | 32 | 25 | 6736 |
|  | % |  | 19.3% | 80.7% | 100.0% |  | 15.9% | 84.1% | 100.0% |  | 90.4% | 5.9% | 2.9% | 0.5% | 0.4% | 100.0% |
| 4 to 6 times | n |  | 611 | 1940 | 2551 |  | 504 | 2047 | 2551 |  | 2226 | 175 | 97 | 27 | 26 | 2551 |
|  | % |  | 24.0% | 76.0% | 100.0% |  | 19.8% | 80.2% | 100.0% |  | 87.3% | 6.9% | 3.8% | 1.1% | 1.0% | 100.0% |
| 1 time per day | n |  | 255 | 825 | 1080 |  | 206 | 874 | 1080 |  | 937 | 82 | 43 | 8 | 10 | 1080 |
|  | % |  | 23.6% | 76.4% | 100.0% |  | 19.1% | 80.9% | 100.0% |  | 86.8% | 7.6% | 4.0% | 0.7% | 0.9% | 100.0% |
| 2 times per day | n |  | 209 | 547 | 756 |  | 168 | 588 | 756 |  | 639 | 63 | 39 | 5 | 10 | 756 |
|  | % |  | 27.6% | 72.4% | 100.0% |  | 22.2% | 77.8% | 100.0% |  | 84.5% | 8.3% | 5.2% | 0.7% | 1.3% | 100.0% |
| 3 times per day | n |  | 100 | 222 | 322 |  | 83 | 239 | 322 |  | 262 | 28 | 19 | 9 | 4 | 322 |
|  | % |  | 31.1% | 68.9% | 100.0% |  | 25.8% | 74.2% | 100.0% |  | 81.4% | 8.7% | 5.9% | 2.8% | 1.2% | 100.0% |
| 4 or more times per day | n |  | 153 | 322 | 475 |  | 123 | 352 | 475 |  | 372 | 33 | 34 | 9 | 27 | 475 |
|  | % |  | 32.2% | 67.8% | 100.0% |  | 25.9% | 74.1% | 100.0% |  | 78.3% | 6.9% | 7.2% | 1.9% | 5.7% | 100.0% |
| Total | n |  | 3557 | 13389 | 16946 |  | 2907 | 14039 | 16946 |  | 15081 | 1057 | 559 | 121 | 128 | 16946 |
|  | % |  | 21.0% | 79.0% | 100.0% |  | 17.2% | 82.8% | 100.0% |  | 89.0% | 6.2% | 3.3% | 0.7% | 0.8% | 100.0% |
|  |  |  | *X^2^* = 124.26, *df* = 6, p < 0.001 | | |  | *X^2^* = 95.59, *df* = 6, p < 0.001 | | |  | *X^2^* = 298.42, *df* = 24, p < 0.001 | | | | | |
| **No milk** |  |  |  |  |  |  |  |  |  |  |  |  |  |  |  |  |
| Did not drink milk | n |  | 1469 | 4471 | 5940 |  | 1202 | 4738 | 5940 |  | 5137 | 450 | 256 | 54 | 43 | 5940 |
|  | % |  | 24.7% | 75.3% | 100.0% |  | 20.2% | 79.8% | 100.0% |  | 86.5% | 7.6% | 4.3% | 0.9% | 0.7% | 100.0% |
| 1 to 3 times | n |  | 1032 | 3727 | 4759 |  | 805 | 3954 | 4759 |  | 4253 | 297 | 145 | 33 | 31 | 4759 |
|  | % |  | 21.7% | 78.3% | 100.0% |  | 16.9% | 83.1% | 100.0% |  | 89.4% | 6.2% | 3.0% | 0.7% | 0.7% | 100.0% |
| 4 to 6 times | n |  | 355 | 1672 | 2027 |  | 293 | 1734 | 2027 |  | 1860 | 100 | 44 | 9 | 14 | 2027 |
|  | % |  | 17.5% | 82.5% | 100.0% |  | 14.5% | 85.5% | 100.0% |  | 91.8% | 4.9% | 2.2% | 0.4% | 0.7% | 100.0% |
| 1 time per day | n |  | 335 | 1716 | 2051 |  | 289 | 1762 | 2051 |  | 1873 | 99 | 56 | 11 | 12 | 2051 |
|  | % |  | 16.3% | 83.7% | 100.0% |  | 14.1% | 85.9% | 100.0% |  | 91.3% | 4.8% | 2.7% | 0.5% | 0.6% | 100.0% |
| 2 times per day | n |  | 208 | 943 | 1151 |  | 174 | 977 | 1151 |  | 1054 | 55 | 25 | 8 | 9 | 1151 |
|  | % |  | 18.1% | 81.9% | 100.0% |  | 15.1% | 84.9% | 100.0% |  | 91.6% | 4.8% | 2.2% | 0.7% | 0.8% | 100.0% |
| 3 times per day | n |  | 68 | 407 | 475 |  | 62 | 413 | 475 |  | 427 | 27 | 17 | 1 | 3 | 475 |
|  | % |  | 14.3% | 85.7% | 100.0% |  | 13.1% | 86.9% | 100.0% |  | 89.9% | 5.7% | 3.6% | .2% | 0.6% | 100.0% |
| 4 or more times per day | n |  | 90 | 453 | 543 |  | 82 | 461 | 543 |  | 477 | 29 | 16 | 5 | 16 | 543 |
|  | % |  | 16.6% | 83.4% | 100.0% |  | 15.1% | 84.9% | 100.0% |  | 87.8% | 5.3% | 2.9% | 0.9% | 2.9% | 100.0% |
| Total | n |  | 3557 | 13389 | 16946 |  | 2907 | 14039 | 16946 |  | 15081 | 1057 | 559 | 121 | 128 | 16946 |
|  | % |  | 21.0% | 79.0% | 100.0% |  | 17.2% | 82.8% | 100.0% |  | 89.0% | 6.2% | 3.3% | 0.7% | 0.8% | 100.0% |
|  |  |  | *X^2^* = 118.15, *df* = 6, p < 0.001 | | |  | *X^2^* = 74.41, *df* = 6, p < 0.001 | | |  | *X^2^* = 120.62, *df* = 24, p < 0.001 | | | | | |
| **Alcohol** |  |  |  |  |  |  |  |  |  |  |  |  |  |  |  |  |
| 0 days | n |  | 2130 | 10903 | 13033 |  | 1708 | 11325 | 13033 |  | 12030 | 639 | 276 | 43 | 45 | 13033 |
|  | % |  | 16.3% | 83.7% | 100.0% |  | 13.1% | 86.9% | 100.0% |  | 92.3% | 4.9% | 2.1% | 0.3% | 0.3% | 100.0% |
| 1 or 2 days | n |  | 717 | 1396 | 2113 |  | 573 | 1540 | 2113 |  | 1721 | 203 | 133 | 31 | 25 | 2113 |
|  | % |  | 33.9% | 66.1% | 100.0% |  | 27.1% | 72.9% | 100.0% |  | 81.4% | 9.6% | 6.3% | 1.5% | 1.2% | 100.0% |
| 3 to 5 days | n |  | 356 | 576 | 932 |  | 290 | 642 | 932 |  | 718 | 113 | 67 | 22 | 12 | 932 |
|  | % |  | 38.2% | 61.8% | 100.0% |  | 31.1% | 68.9% | 100.0% |  | 77.0% | 12.1% | 7.2% | 2.4% | 1.3% | 100.0% |
| 6 to 9 days | n |  | 175 | 288 | 463 |  | 164 | 299 | 463 |  | 351 | 52 | 45 | 8 | 7 | 463 |
|  | % |  | 37.8% | 62.2% | 100.0% |  | 35.4% | 64.6% | 100.0% |  | 75.8% | 11.2% | 9.7% | 1.7% | 1.5% | 100.0% |
| 10 to 19 days | n |  | 101 | 143 | 244 |  | 99 | 145 | 244 |  | 181 | 28 | 17 | 8 | 10 | 244 |
|  | % |  | 41.4% | 58.6% | 100.0% |  | 40.6% | 59.4% | 100.0% |  | 74.2% | 11.5% | 7.0% | 3.3% | 4.1% | 100.0% |
| 20 to 29 days | n |  | 30 | 28 | 58 |  | 30 | 28 | 58 |  | 28 | 10 | 9 | 6 | 5 | 58 |
|  | % |  | 51.7% | 48.3% | 100.0% |  | 51.7% | 48.3% | 100.0% |  | 48.3% | 17.2% | 15.5% | 10.3% | 8.6% | 100.0% |
| All 30 days | n |  | 48 | 55 | 103 |  | 43 | 60 | 103 |  | 52 | 12 | 12 | 3 | 24 | 103 |
|  | % |  | 46.6% | 53.4% | 100.0% |  | 41.7% | 58.3% | 100.0% |  | 50.5% | 11.7% | 11.7% | 2.9% | 23.3% | 100.0% |
| Total | n |  | 3557 | 13389 | 16946 |  | 2907 | 14039 | 16946 |  | 15081 | 1057 | 559 | 121 | 128 | 16946 |
|  | % |  | 21.0% | 79.0% | 100.0% |  | 17.2% | 82.8% | 100.0% |  | 89.0% | 6.2% | 3.3% | 0.7% | 0.8% | 100.0% |
|  |  |  | *X^2^* = 763.41, *df* = 6, p < 0.001 | | |  | *X^2^* = 721.27, *df* = 6, p < 0.001 | | |  | *X^2^* = 1539.99, *df* = 24, p < 0.001 | | | | | |
| **Cigarette** |  |  |  |  |  |  |  |  |  |  |  |  |  |  |  |  |
| 0 days | n |  | 3213 | 13051 | 16264 |  | 2586 | 13678 | 16264 |  | 14690 | 940 | 464 | 86 | 84 | 16264 |
|  | % |  | 19.8% | 80.2% | 100.0% |  | 15.9% | 84.1% | 100.0% |  | 90.3% | 5.8% | 2.9% | 0.5% | 0.5% | 100.0% |
| 1 or 2 days | n |  | 150 | 158 | 308 |  | 137 | 171 | 308 |  | 191 | 53 | 45 | 14 | 5 | 308 |
|  | % |  | 48.7% | 51.3% | 100.0% |  | 44.5% | 55.5% | 100.0% |  | 62.0% | 17.2% | 14.6% | 4.5% | 1.6% | 100.0% |
| 3 to 5 days | n |  | 53 | 68 | 121 |  | 55 | 66 | 121 |  | 80 | 19 | 16 | 2 | 4 | 121 |
|  | % |  | 43.8% | 56.2% | 100.0% |  | 45.5% | 54.5% | 100.0% |  | 66.1% | 15.7% | 13.2% | 1.7% | 3.3% | 100.0% |
| 6 to 9 days | n |  | 31 | 18 | 49 |  | 29 | 20 | 49 |  | 20 | 15 | 10 | 3 | 1 | 49 |
|  | % |  | 63.3% | 36.7% | 100.0% |  | 59.2% | 40.8% | 100.0% |  | 40.8% | 30.6% | 20.4% | 6.1% | 2.0% | 100.0% |
| 10 to 19 days | n |  | 40 | 31 | 71 |  | 39 | 32 | 71 |  | 37 | 13 | 7 | 10 | 4 | 71 |
|  | % |  | 56.3% | 43.7% | 100.0% |  | 54.9% | 45.1% | 100.0% |  | 52.1% | 18.3% | 9.9% | 14.1% | 5.6% | 100.0% |
| 20 to 29 days | n |  | 13 | 10 | 23 |  | 14 | 9 | 23 |  | 11 | 3 | 6 | 0 | 3 | 23 |
|  | % |  | 56.5% | 43.5% | 100.0% |  | 60.9% | 39.1% | 100.0% |  | 47.8% | 13.0% | 26.1% | 0.0% | 13.0% | 100.0% |
| All 30 days | n |  | 57 | 53 | 110 |  | 47 | 63 | 110 |  | 52 | 14 | 11 | 6 | 27 | 110 |
|  | % |  | 51.8% | 48.2% | 100.0% |  | 42.7% | 57.3% | 100.0% |  | 47.3% | 12.7% | 10.0% | 5.5% | 24.5% | 100.0% |
| Total | n |  | 3557 | 13389 | 16946 |  | 2907 | 14039 | 16946 |  | 15081 | 1057 | 559 | 121 | 128 | 16946 |
|  | % |  | 21.0% | 79.0% | 100.0% |  | 17.2% | 82.8% | 100.0% |  | 89.0% | 6.2% | 3.3% | 0.7% | 0.8% | 100.0% |
|  |  |  | *X^2^* = 382.38, *df* = 6, p < 0.001 | | |  | *X^2^* = 461.76, *df* = 6, p < 0.001 | | |  | *X^2^* = 1733.73, *df* = 24, p < 0.001 | | | | | |
| **Electronic vapor** |  |  |  |  |  |  |  |  |  |  |  |  |  |  |  |  |
| 0 days | n |  | 2233 | 11476 | 13709 |  | 1783 | 11926 | 13709 |  | 12687 | 658 | 268 | 44 | 52 | 13709 |
|  | % |  | 16.3% | 83.7% | 100.0% |  | 13.0% | 87.0% | 100.0% |  | 92.5% | 4.8% | 2.0% | 0.3% | 0.4% | 100.0% |
| 1 or 2 days | n |  | 239 | 506 | 745 |  | 205 | 540 | 745 |  | 608 | 75 | 47 | 8 | 7 | 745 |
|  | % |  | 32.1% | 67.9% | 100.0% |  | 27.5% | 72.5% | 100.0% |  | 81.6% | 10.1% | 6.3% | 1.1% | 0.9% | 100.0% |
| 3 to 5 days | n |  | 162 | 236 | 398 |  | 137 | 261 | 398 |  | 310 | 46 | 24 | 13 | 5 | 398 |
|  | % |  | 40.7% | 59.3% | 100.0% |  | 34.4% | 65.6% | 100.0% |  | 77.9% | 11.6% | 6.0% | 3.3% | 1.3% | 100.0% |
| 6 to 9 days | n |  | 110 | 176 | 286 |  | 106 | 180 | 286 |  | 199 | 40 | 37 | 4 | 6 | 286 |
|  | % |  | 38.5% | 61.5% | 100.0% |  | 37.1% | 62.9% | 100.0% |  | 69.6% | 14.0% | 12.9% | 1.4% | 2.1% | 100.0% |
| 10 to 19 days | n |  | 179 | 256 | 435 |  | 151 | 284 | 435 |  | 321 | 58 | 40 | 11 | 5 | 435 |
|  | % |  | 41.1% | 58.9% | 100.0% |  | 34.7% | 65.3% | 100.0% |  | 73.8% | 13.3% | 9.2% | 2.5% | 1.1% | 100.0% |
| 20 to 29 days | n |  | 166 | 215 | 381 |  | 139 | 242 | 381 |  | 293 | 43 | 33 | 8 | 4 | 381 |
|  | % |  | 43.6% | 56.4% | 100.0% |  | 36.5% | 63.5% | 100.0% |  | 76.9% | 11.3% | 8.7% | 2.1% | 1.0% | 100.0% |
| All 30 days | n |  | 468 | 524 | 992 |  | 386 | 606 | 992 |  | 663 | 137 | 110 | 33 | 49 | 992 |
|  | % |  | 47.2% | 52.8% | 100.0% |  | 38.9% | 61.1% | 100.0% |  | 66.8% | 13.8% | 11.1% | 3.3% | 4.9% | 100.0% |
| Total | n |  | 3557 | 13389 | 16946 |  | 2907 | 14039 | 16946 |  | 15081 | 1057 | 559 | 121 | 128 | 16946 |
|  | % |  | 21.0% | 79.0% | 100.0% |  | 17.2% | 82.8% | 100.0% |  | 89.0% | 6.2% | 3.3% | 0.7% | 0.8% | 100.0% |
|  |  |  | *X^2^* = 1017.80, *df* = 6, p < 0.001 | | |  | *X^2^* = 910.49, *df* = 6, p < 0.001 | | |  | *X^2^* = 1270.69, *df* = 24, p < 0.001 | | | | | |
| **Marijuana** |  |  |  |  |  |  |  |  |  |  |  |  |  |  |  |  |
| 0 times | n |  | 2423 | 11848 | 14271 |  | 1937 | 12334 | 14271 |  | 13110 | 735 | 308 | 59 | 59 | 14271 |
|  | % |  | 17.0% | 83.0% | 100.0% |  | 13.6% | 86.4% | 100.0% |  | 91.9% | 5.2% | 2.2% | 0.4% | 0.4% | 100.0% |
| 1 or 2 times | n |  | 341 | 538 | 879 |  | 269 | 610 | 879 |  | 699 | 92 | 60 | 15 | 13 | 879 |
|  | % |  | 38.8% | 61.2% | 100.0% |  | 30.6% | 69.4% | 100.0% |  | 79.5% | 10.5% | 6.8% | 1.7% | 1.5% | 100.0% |
| 3 to 9 times | n |  | 246 | 290 | 536 |  | 218 | 318 | 536 |  | 386 | 71 | 57 | 15 | 7 | 536 |
|  | % |  | 45.9% | 54.1% | 100.0% |  | 40.7% | 59.3% | 100.0% |  | 72.0% | 13.2% | 10.6% | 2.8% | 1.3% | 100.0% |
| 10 to 19 times | n |  | 148 | 209 | 357 |  | 133 | 224 | 357 |  | 254 | 48 | 35 | 8 | 12 | 357 |
|  | % |  | 41.5% | 58.5% | 100.0% |  | 37.3% | 62.7% | 100.0% |  | 71.1% | 13.4% | 9.8% | 2.2% | 3.4% | 100.0% |
| 20 to 39 times | n |  | 143 | 178 | 321 |  | 121 | 200 | 321 |  | 231 | 34 | 40 | 13 | 3 | 321 |
|  | % |  | 44.5% | 55.5% | 100.0% |  | 37.7% | 62.3% | 100.0% |  | 72.0% | 10.6% | 12.5% | 4.0% | 0.9% | 100.0% |
| 40 or more times | n |  | 256 | 326 | 582 |  | 229 | 353 | 582 |  | 401 | 77 | 59 | 11 | 34 | 582 |
|  | % |  | 44.0% | 56.0% | 100.0% |  | 39.3% | 60.7% | 100.0% |  | 68.9% | 13.2% | 10.1% | 1.9% | 5.8% | 100.0% |
| Total | n |  | 3557 | 13389 | 16946 |  | 2907 | 14039 | 16946 |  | 15081 | 1057 | 559 | 121 | 128 | 16946 |
|  | % |  | 21.0% | 79.0% | 100.0% |  | 17.2% | 82.8% | 100.0% |  | 89.0% | 6.2% | 3.3% | 0.7% | 0.8% | 100.0% |
|  |  |  | *X^2^* = 890.13, *df* = 5, p < 0.001 | | |  | *X^2^* = 847.74, *df* = 5, p < 0.001 | | |  | *X^2^* = 1053.20, *df* = 20, p < 0.001 | | | | | |
| **Prescription pain medicine** |  |  |  |  |  |  |  |  |  |  |  |  |  |  |  |  |
| 0 times | n |  | 3077 | 12849 | 15926 |  | 2481 | 13445 | 15926 |  | 14428 | 902 | 427 | 86 | 83 | 15926 |
|  | % |  | 19.3% | 80.7% | 100.0% |  | 15.6% | 84.4% | 100.0% |  | 90.6% | 5.7% | 2.7% | 0.5% | 0.5% | 100.0% |
| 1 or 2 times | n |  | 233 | 284 | 517 |  | 189 | 328 | 517 |  | 355 | 82 | 57 | 17 | 6 | 517 |
|  | % |  | 45.1% | 54.9% | 100.0% |  | 36.6% | 63.4% | 100.0% |  | 68.7% | 15.9% | 11.0% | 3.3% | 1.2% | 100.0% |
| 3 to 9 times | n |  | 130 | 139 | 269 |  | 121 | 148 | 269 |  | 174 | 41 | 37 | 9 | 8 | 269 |
|  | % |  | 48.3% | 51.7% | 100.0% |  | 45.0% | 55.0% | 100.0% |  | 64.7% | 15.2% | 13.8% | 3.3% | 3.0% | 100.0% |
| 10 to 19 times | n |  | 50 | 51 | 101 |  | 44 | 57 | 101 |  | 60 | 18 | 17 | 5 | 1 | 101 |
|  | % |  | 49.5% | 50.5% | 100.0% |  | 43.6% | 56.4% | 100.0% |  | 59.4% | 17.8% | 16.8% | 5.0% | 1.0% | 100.0% |
| 20 to 39 times | n |  | 21 | 18 | 39 |  | 26 | 13 | 39 |  | 20 | 5 | 11 | 1 | 2 | 39 |
|  | % |  | 53.8% | 46.2% | 100.0% |  | 66.7% | 33.3% | 100.0% |  | 51.3% | 12.8% | 28.2% | 2.6% | 5.1% | 100.0% |
| 40 or more times | n |  | 46 | 48 | 94 |  | 46 | 48 | 94 |  | 44 | 9 | 10 | 3 | 28 | 94 |
|  | % |  | 48.9% | 51.1% | 100.0% |  | 48.9% | 51.1% | 100.0% |  | 46.8% | 9.6% | 10.6% | 3.2% | 29.8% | 100.0% |
| Total | n |  | 3557 | 13389 | 16946 |  | 2907 | 14039 | 16946 |  | 15081 | 1057 | 559 | 121 | 128 | 16946 |
|  | % |  | 21.0% | 79.0% | 100.0% |  | 17.2% | 82.8% | 100.0% |  | 89.0% | 6.2% | 3.3% | 0.7% | 0.8% | 100.0% |
|  |  |  | *X^2^* = 447.88, *df* = 5, p < 0.001 | | |  | *X^2^* = 495.01, *df* = 5, p < 0.001 | | |  | *X^2^* = 1778.79, *df* = 20, p < 0.001 | | | | | |
| **Sad or hopeless** |  |  |  |  |  |  |  |  |  |  |  |  |  |  |  |  |
| Yes | n |  | 3086 | 3609 | 6695 |  | 2496 | 4199 | 6695 |  | 5220 | 762 | 493 | 109 | 111 | 6695 |
|  | % |  | 46.1% | 53.9% | 100.0% |  | 37.3% | 62.7% | 100.0% |  | 78.0% | 11.4% | 7.4% | 1.6% | 1.7% | 100.0% |
| No | n |  | 471 | 9780 | 10251 |  | 411 | 9840 | 10251 |  | 9861 | 295 | 66 | 12 | 17 | 10251 |
|  | % |  | 4.6% | 95.4% | 100.0% |  | 4.0% | 96.0% | 100.0% |  | 96.2% | 2.9% | .6% | 0.1% | 0.2% | 100.0% |
| Total | n |  | 3557 | 13389 | 16946 |  | 2907 | 14039 | 16946 |  | 15081 | 1057 | 559 | 121 | 128 | 16946 |
|  | % |  | 21.0% | 79.0% | 100.0% |  | 17.2% | 82.8% | 100.0% |  | 89.0% | 6.2% | 3.3% | 0.7% | 0.8% | 100.0% |
|  |  |  | *X^2^* = 4205.68, *df* = 1*, p < 0.001* | | |  | *X^2^* = 3154.75, *df* = 1, p < 0.001 | | |  | *X^2^* = 1424.01, *df* = 4, p < 0.001 | | | | | |
| **Mental health** |  |  |  |  |  |  |  |  |  |  |  |  |  |  |  |  |
| Never | n |  | 154 | 3765 | 3919 |  | 134 | 3785 | 3919 |  | 3730 | 133 | 34 | 10 | 12 | 3919 |
|  | % |  | 3.9% | 96.1% | 100.0% |  | 3.4% | 96.6% | 100.0% |  | 95.2% | 3.4% | .9% | 0.3% | 0.3% | 100.0% |
| Rarely | n |  | 273 | 3359 | 3632 |  | 255 | 3377 | 3632 |  | 3435 | 124 | 45 | 17 | 11 | 3632 |
|  | % |  | 7.5% | 92.5% | 100.0% |  | 7.0% | 93.0% | 100.0% |  | 94.6% | 3.4% | 1.2% | 0.5% | 0.3% | 100.0% |
| Sometimes | n |  | 842 | 3813 | 4655 |  | 675 | 3980 | 4655 |  | 4276 | 253 | 99 | 18 | 9 | 4655 |
|  | % |  | 18.1% | 81.9% | 100.0% |  | 14.5% | 85.5% | 100.0% |  | 91.9% | 5.4% | 2.1% | 0.4% | 0.2% | 100.0% |
| Most of the time | n |  | 1384 | 1882 | 3266 |  | 1075 | 2191 | 3266 |  | 2645 | 356 | 194 | 40 | 31 | 3266 |
|  | % |  | 42.4% | 57.6% | 100.0% |  | 32.9% | 67.1% | 100.0% |  | 81.0% | 10.9% | 5.9% | 1.2% | 0.9% | 100.0% |
| Always | n |  | 904 | 570 | 1474 |  | 768 | 706 | 1474 |  | 995 | 191 | 187 | 36 | 65 | 1474 |
|  | % |  | 61.3% | 38.7% | 100.0% |  | 52.1% | 47.9% | 100.0% |  | 67.5% | 13.0% | 12.7% | 2.4% | 4.4% | 100.0% |
| Total | n |  | 3557 | 13389 | 16946 |  | 2907 | 14039 | 16946 |  | 15081 | 1057 | 559 | 121 | 128 | 16946 |
|  | % |  | 21.0% | 79.0% | 100.0% |  | 17.2% | 82.8% | 100.0% |  | 89.0% | 6.2% | 3.3% | 0.7% | 0.8% | 100.0% |
|  |  |  | *X^2^* = 3456.01, *df* = 4, p < 0.001 | | |  | *X^2^* = 2643.38, *df* = 4, p < 0.001 | | |  | *X^2^* = 1452.89, *df* = 16, p < 0.001 | | | | | |
